# LncRNA Sox2OT-V7 promotes doxorubicin-induced autophagy and chemoresistance in osteosarcoma via tumor-suppressive miR-142/miR-22

**DOI:** 10.18632/aging.103004

**Published:** 2020-04-16

**Authors:** Kewei Zhu, Yang Yuan, Jie Wen, Ding Chen, Weihong Zhu, Zhengxiao Ouyang, Wanchun Wang

**Affiliations:** 1 Department of Orthopedics, The Second Xiangya Hospital, Central South University, Changsha, Hunan 410011, China; 2 Department of Orthopedics, Xiangya Changde Hospital, Changde, Hunan 415000, China; 3 Department of Pediatric Orthopedics, Hunan Provincial Peoples’ Hospital, Changsha, Hunan 410006, China

**Keywords:** osteosarcoma (OS), doxorubicin (Dox), autophagy, SOX2 overlapping transcript lncRNA transcript variant 7 (Sox2OT-V7), chemoresistance

## Abstract

Doxorubicin (Dox) is one of the most commonly used chemotherapeutic drugs for osteosarcoma (OS) treatment. In the present study, we attempted to investigate the mechanism by which Sox2OT-V7 dysregulation affects Dox chemoresistance to provide a novel experimental basis for developing neoadjuvant therapy. Sox2OT-V7 expression is upregulated in OS tissues, particularly in chemoresistant OS tissues, and in OS cell lines compared to controls. Dox treatment induces autophagy and Sox2OT-V7 expression in U2OS cells, and Dox-induced autophagy is partially attenuated by Sox2OT-V7 silencing. Knocking down Sox2OT-V7 or blocking autophagy in Dox-resistant U2OS/Dox cells resensitizes the cells to Dox treatment *in vitro*. Moreover, Sox2OT-V7 directly targets miR-142/miR-22 to inhibit their expression, and the effect of Sox2OT-V7 silencing on U2OS cell autophagy and U2OS/Dox cell sensitivity to Dox can be reversed by miR-142/miR-22 inhibition. Sox2OT-V7 silencing enhances the suppressive effects of Dox on U2OS/Dox cell-derived tumor growth *in vivo*, while miR-22 inhibition or miR-142 inhibition reverses the effects of Sox2OT-V7 silencing on Dox-induced suppression on tumor growth. Finally, miR-142 directly targets ULK1, ATG4A, and ATG5, while miR-22 directly targets ULK1 to inhibit the expression of the target gene; The Sox2OT-V7/miR-142/miR-22 axis modulates autophagy in OS cells by regulating ULK1, ATG4A, and ATG5.

## INTRODUCTION

Osteosarcoma (OS) is one of the most common primary bone tumors in children and adolescents, OS originates from the malignant transformation of mesenchymal cells and has a high mortality rate [1]. Doxorubicin (Dox) is one of the first line chemotherapeutic drugs for OS [2]. However, the use of Dox at a low-dose not only reduces its effectiveness but also leads to drug resistance, while dose increase would cause severe cardiotoxicity [3], leading to limits on clinical application. Therefore, there is an urgent need to investigate the mechanism of Dox chemoresistance and to develop neoadjuvant therapy.

Dox treatment can induce pro-survival autophagy in cells [4–6]. Autophagy is the primary pathway involved in the degradation of proteins and organelles, cellular remodeling, and survival during nutrient starvation [7]. Autophagy is a critical regulator of cellular homeostasis, promoting the controlled degradation of cytoplasmic material both at steady-state and during nutrient deprivation [8]. Studies have shown the critical roles of autophagy in OS chemoresistance to Dox [9, 10]. In our previous study, we also revealed that Epigallocatechin gallate (EGCG) reduced doxorubicin-induced pro-survival autophagy by decreasing Sox2OT (SOX2 overlapping transcript) lncRNA transcript variant 7 (Sox2OT-V7) to improve the growth inhibition of Dox [11]. Since lncRNA Sox2OT-V7 is overexpressed in OS tissues and cell lines, investigating the specific function and mechanism of Sox2OT-V7 in autophagy and the resistance of OS cells to Dox may provide new directions for combating OS chemoresistance.

LncRNAs can exert their biological functions by serving as sponges for miRNAs to counteract miRNA-mediated suppression of miRNA downstream targets [12]. To investigate the molecular mechanism of Sox2OT-V7 in Dox-induced autophagy in OS, we searched TCGA online data for candidate miRNAs related to Sox2OT-V7 and autophagy. As shown in Supplementary Table 2, some miRNA, including miR-22 and miR-142, were negatively correlated with Sox2OT-V7 expression in OS (Supplementary Figure 1). miR-22 regulates 5-FU sensitivity by inhibiting autophagy and promoting apoptosis in colorectal cancer cells [13], while miR-142 targets ATG5/ATG16L1 to sensitize hepatocellular carcinoma cells to sorafenib by inactivating autophagy [14]. Moreover, online tools indicate that Sox2OT-V7 may target miR-142 and miR-22. Thus, we hypothesize that Sox2OT-V7 may exert its effect on OS by regulating miR-142 and miR-22.

In the present study, the expression of Sox2OT-V7 in OS tissue samples and cell lines was examined. The effect of Sox2OT-V7 on autophagy-related markers and autophagy flux was evaluated. The effects of Sox2OT-V7 silencing on Dox-resistant U2OS/Dox cell viability and U2OS/Dox cell-derived tumor growth were also examined. Next, the predicted binding between Sox2OT-V7 and miR-142/miR-22, between miR-142/miR-22 and ULK1, ATG4A, and ATG5 was validated. In addition, the dynamic effect of Sox2OT-V7 and miR-142/miR-22 on Dox suppression on U2OS/Dox cell viability, and the dynamic effect of Sox2OT-V7, miR-142/miR-22, ULK1, ATG4A, and ATG5 on autophagy-related genes and autophagy flux was evaluated. Finally, the expression of these factors and their correlations in tissue samples were determined. In summary, we provide a novel mechanism by which Sox2OT-V7 modulates autophagy in OS cells, therefore affects chemoresistance to Dox-based therapy from the perspective of lncRNA-miRNA-mRNA regulation.

## RESULTS

### Expression of Sox2OT-V7 in tissue samples and cell lines and its correlation with autophagy markers

**Figure 1:**
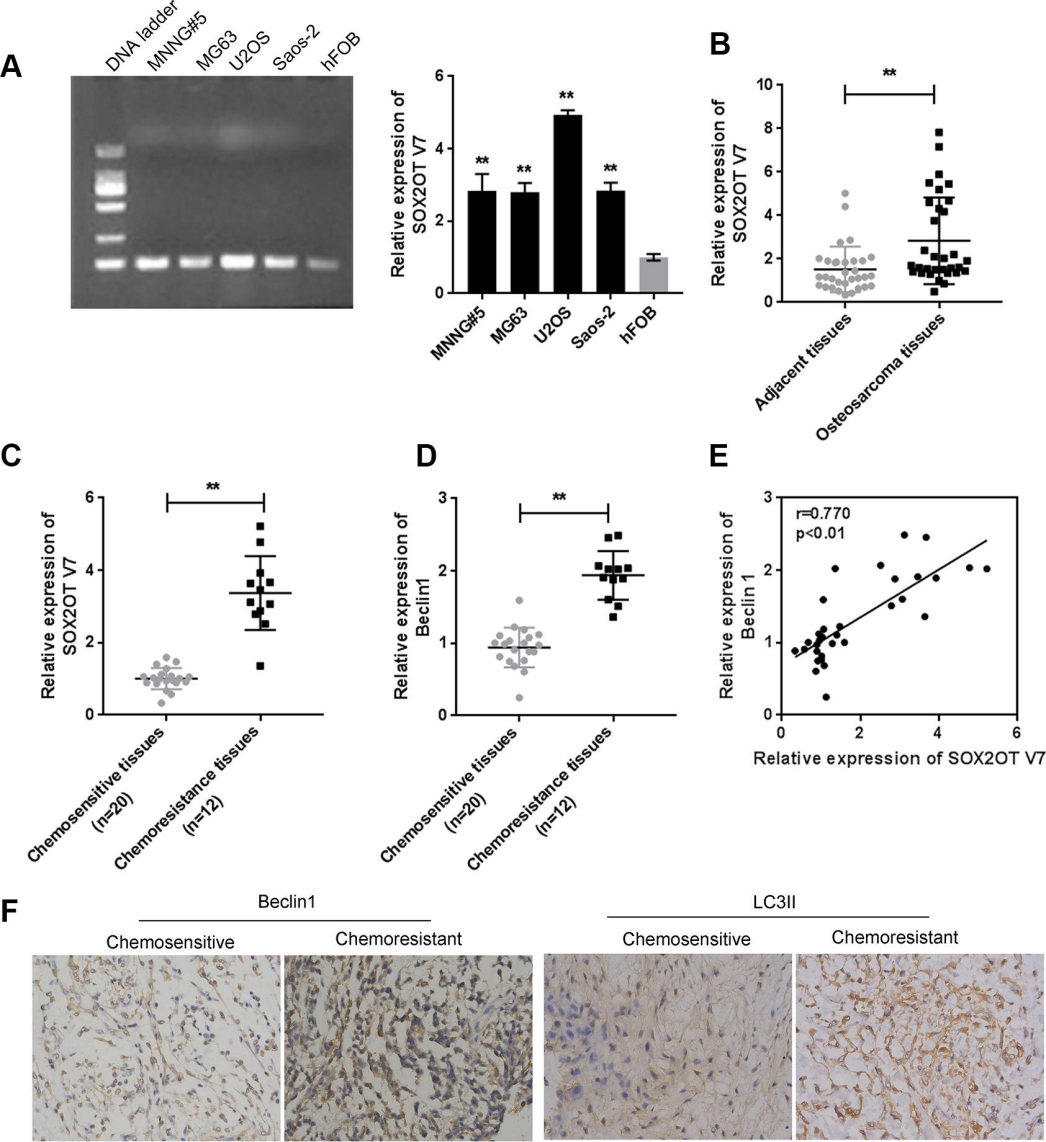
**Expression of Sox2OT-V7 in tissue samples and cell lines and its correlation with autophagy markers.** (**A**) Sox2OT-V7 expression in four OS cell lines and a normal osteoblast cell line was determined by qPCR. (**B**) Sox2OT-V7 expression in 32 paired OS and nontumorous tissue samples was determined by qPCR. (**C**, **D**) Sox2OT-V7, and Beclin 1 expression in 20 chemosensitive and 12 chemoresistant OS tissues was determined by qPCR. (**E**) The correlation Sox2OT-V7 and Beclin 1 mRNA expression in tissue samples was analyzed by Pearson’s correlation analysis. (**F**) The protein content and localization of Beclin 1 and LC3II in tissue samples was revealed by IHC staining. The data are presented as mean ± SD of three independent experiments. *P<0.05, **P<0.01.

Since we have revealed that Sox2OT-V7 contributes to the chemoresistance of OS to doxorubicin via autophagy [11], in the present study, the expression of Sox2OT-V7 was first examined in tissue samples and cell lines. Consistent with our previous research, Sox2OT-V7 expression was significantly upregulated in OS cell lines, compared to a normal osteoblast cell line, hFOB (Figure 1A), and expression was most upregulated in U2OS cells. Similarly, Sox2OT-V7 expression was upregulated in OS tissue samples, compared to noncancerous tissue samples (Figure 1B) and was higher in chemo-resistant tissues than in chemosensitive tissues (Figure 1C).

As for autophagy markers, the mRNA level of Beclin 1 was higher in chemoresistant tissues than in chemosensitive tissues (Figure 1D). IHC staining showed that the Beclin 1 and LC3II levels were increased in chemoresistant tissues (Figure 1F). Moreover, Sox2OT-V7 expression was positively correlated with Beclin 1 (Figure 1E). These data indicate that Sox2OT-V7 is overexpressed in OS, and positively correlated with autophagy markers.

### Dox treatment induces autophagy and Sox2OT-V7 expression in U2OS cells

**Figure 2:**
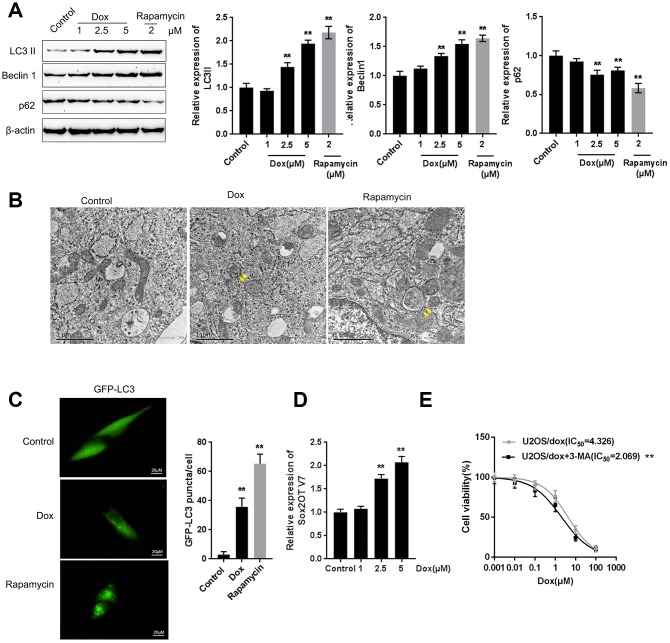
**Dox treatment induces autophagy and Sox2OT-V7 expression in U2OS cells.** (**A**) U2OS cells were treated with 1, 2.5, or 5 μM Dox or 2 μM rapamycin, and the expression of LC3II, Beclin 1, and p62 was measured. U2OS cells with stable eGFP-LC3 expression were treated with Dox (5 μM) or rapamycin (2 μM) for 24 h. Autophagy was examined by transmission electron microscopy (**B**) puncta were imaged by using a confocal microscope, and representative images are presented (**C**). (**D**) U2OS cells were treated with 1, 2.5 or 5 μM Dox and examined for the expression of Sox2OT-V7. (**E**) U2OS cells were treated with 0.001, 0.01, 0.1, 1, 10, and 100 μM Dox in the presence or absence of the autophagy inhibitor 3-MA and examined for cell viability (IC_50_ value). The data are presented as the mean ± SD of three independent experiments. **P<0.01.

Next, we examined whether Dox induces autophagy and Sox2OT-V7 expression in U2OS cells. U2OS cells were treated with Dox (1, 2.5, and 5 μM) or rapamycin (2 μM), a representative autophagic agonist, and examined for autophagy markers, autophagy flux, and Sox2OT-V7 expression. As shown by immunoblotting, Dox significantly increased LC3II and Beclin 1 protein levels in a dose-dependent manner (Figure 2A). The inducible effect of 5 μM Dox on autophagy was close to that of 2 μM rapamycin (Figure 2A). Simultaneously, the protein levels of autophagy substrate p62 were significantly decreased by 2.5 and 5 μM Dox and 2 μM rapamycin (Figure 2A), indicating that autophagy was enhanced by 2.5 and 5 μM Dox treatment and 2 μM rapamycin.

U2OS cells with stable eGFP-LC3 expression were used to confirm Dox-induced autophagy using rapamycin treatment as a positive control. Figure 2B shows that the autophagy in U2OS cells was increased by Dox and rapamycin treatment. As shown in Figure 2C, green puncta were increased after exposure to Dox in U2OS cells with stable eGFP-LC3 expression; this result was similar to that of rapamycin. Moreover, Sox2OT-V7 expression was induced by Dox treatment in a concentration-dependent manner (Figure 2D), further indicating that Dox treatment induced autophagy and Sox2OT-V7 expression in U2OS cells.

Autophagy might be one mechanism by which neoplastic cells maintain survival upon chemotherapy treatment. Under such conditions, blocking autophagy could trigger apoptosis, therefore enhancing the curative effects of these chemotherapies and reducing chemoresistance [15, 16]. Here, we constructed a Dox-resistant U2OS cell line, U2OS/Dox, by treating original U2OS cells with increasing concentrations of Dox (data not shown). Next, we treated U2OS/Dox cells with a series of concentrations of Dox (0.001, 0.01, 0.1, 1, 10, and 100 μM) in the presence or absence of the autophagy inhibitor 3-MA and examined cell viability to investigate whether the autophagy inhibitor would affect the effects of Dox on U2OS cells. As shown in Figure 2E, 3-MA treatment significantly (**P<0.01) enhanced the suppressive effects of Dox on U2OS cell viability, as manifested as the IC_50_ values reduced from 4.326 to 2.069 μM. These data suggest that autophagy inhibitors might resensitize U2OS cells to Dox treatment.

**Figure 3:**
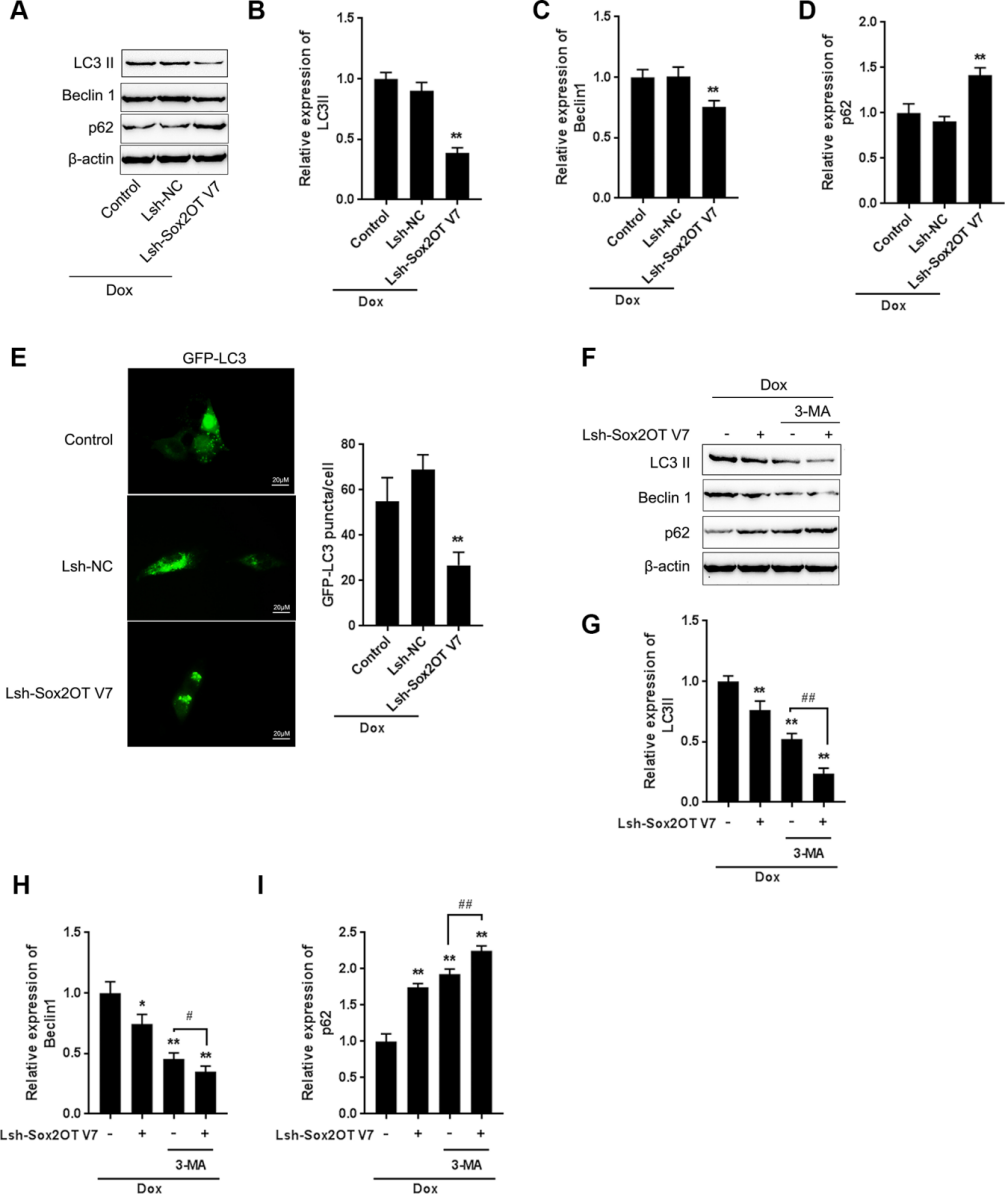
**LncRNA Sox2OT-V7 silencing attenuates Dox-induced autophagy in OS cells.** (**A**–**D**) Sox2OT-V7 silencing in U2OS cells was achieved by infection with Lsh-Sox2OT-V7. Sox2OT-V7-silenced U2OS cells were treated with Dox, and examined for the protein levels of LC3II, Beclin 1, and p62 were examined. (**E**) Sox2OT-V7-silenced U2OS cells with stable eGFP-LC3 expression were treated with Dox (5 μM) for 24 h, and the formation of puncta was examined by using a confocal microscope. Representative images are presented. (**F**–**I**) Sox2OT-V7-silenced U2OS cells were cotreated with Dox (5 μM) and 3-MA (5 μM) for 24 h, and he protein levels of LC3 II, Beclin 1, and p62 were examined. The data are presented as the mean ± SD of three independent experiments. *P<0.05, **P<0.01, compared to the control group; #*P*<0.05, ##*P*<0.01, compared to the Dox group.

### LncRNA Sox2OT-V7 silencing attenuates Dox-induced autophagy in OS cells

To investigate the specific function of Sox2OT-V7 on Dox-induced autophagy in OS cells, we achieved Sox2OT-V7 silencing by infecting cells with Lsh- Sox2OT-V7, and confirmed the efficiency by qPCR (Supplementary Figure 1). Next, Sox2OT-V7 silenced U2OS cells were treated with Dox and examined for autophagy-related factors. As shown by immunoblotting, Dox-induced LC3II and Beclin 1 protein levels were both significantly decreased, while Dox-suppressed p62 protein was partially rescued by Sox2OT-V7 silencing (Figure 3A–3D). Consistent results were observed in OS cells with stable eGFP-LC3 expression, in which the green puncta were reduced by Sox2OT-V7 silencing (Figure 3E).

To further confirm the function of Sox2OT-V7 on Dox-induced autophagy in U2OS cells, we cotreated Sox2OT-V7 silenced U2OS cells with Dox and 3-MA and then examined autophagy-related factors. As revealed by the protein levels of LC3II, Beclin 1 and p62, Dox-induced autophagy in U2OS cells could be partially attenuated by Sox2OT-V7 silencing or 3-MA treatment alone and markedly suppressed by a combination of Sox2OT-V7 silencing and 3-MA treatment (Figure 3F–3I). These data indicate that Sox2OT-V7 silencing serves as an autophagy inhibitor in U2OS cells.

### LncRNA Sox2OT-V7 silencing promotes the sensitivity of OS cells to Dox

**Figure 4:**
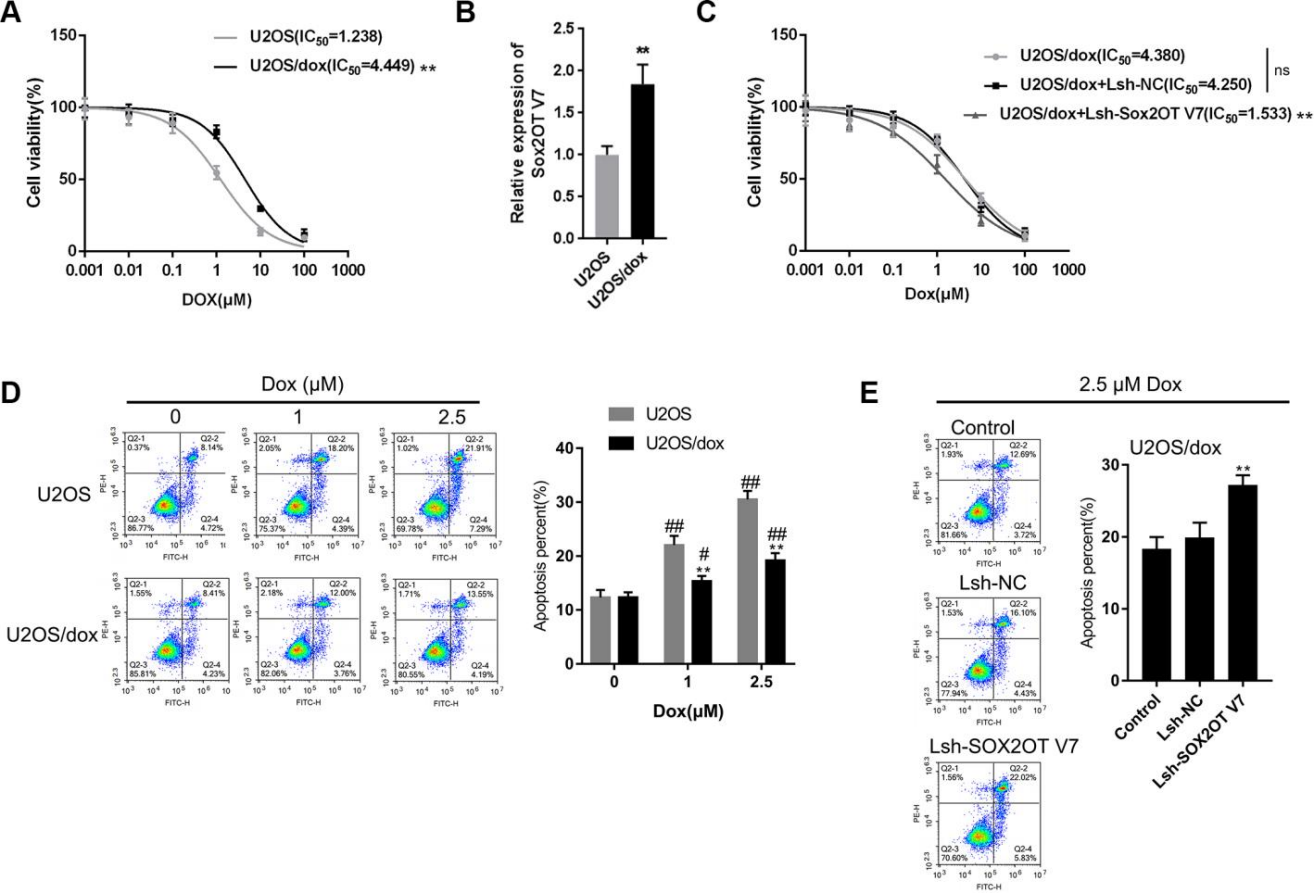
**LncRNA Sox2OT-V7 silencing increases the sensitivity of OS cells to Dox.** (**A**) Parantal U2OS cells and Dox-resistant U2OS/Dox cells were treated with a series of concentrations of Dox (0.001, 0,01, 0.1, 1, 10, and 100 μM) and examined for cell viability by MTT assay. (**B**) The expression of lncRNA Sox2OT-V7 in original U2OS and Dox-resistant U2OS/Dox cells was determined using real-time PCR. (**C**) U2OS/Dox cells were transfected with Lsh-NC or Lsh-Sox2OT-V7, treated with a series of concentrations of Dox (0.001, 0,01, 0.1, 1, 10, and 100 μM), and examined for cell viability by MTT assay. (**D**) Parental U2OS and Dox-resistant U2OS/Dox cells were treated with a series of concentrations of Dox (0, 1, and 2.5 μM) and cell apoptosis was examined by flow cytometry. **P<0.01, compared to the U2OS group; #*P*<0.05, ##*P*<0.01, compared to the 0 μM Dox group. (**E**) U2OS/Dox cells were transfected with Lsh-NC or Lsh-Sox2OT-V7, treated with 2.5 μM Dox, and examined for cell apoptosis by flow cytometry. **P<0.01, compared to U2OS or Lsh-NC group; ##*P*<0.01, compared to 0 μM Dox group.

After confirming that Sox2OT-V7 silencing could block autophagy in U2OS cells, we continued to investigate whether lncRNA Sox2OT-V7 silencing could resensitize OS cells to Dox treatment. As shown in Figure 4A, under Dox treatment, the IC_50_ value of the original U2OS was 1.238 μM, while that of U2OS/Dox was upregulated to 4.449 μM, indicating that U2OS/Dox cells are resistant to Dox treatment. In U2OS/Dox cells, the expression of lncRNA Sox2OT-V7 was significantly upregulated, compared to that in the original U2OS cells (Figure 4B). After generating lncRNA Sox2OT-V7 silencing in U2OS/Dox cells, the suppressive effects of Dox on U2OS/Dox cell viability were significantly (**P<0.01) enhanced, as manifested by a reduced IC_50_ value: 4.380 μM for non-transfected U2OS/Dox cells, 4.250 for Lsh-NC-transfected U2OS/Dox cells, and 1.533 for Lsh-Sox2OT-V7-transfected U2OS/Dox cells (Figure 4C). Moreover, the apoptosis percentage of U2OS/Dox cells was significantly lower than that of the original U2OS cells (Figure 4D). After silencing lncRNA Sox2OT-V7 in U2OS/Dox cells, the apoptosis rate was increased compared to that in the non-transfected cells and Lsh-NC-transfected cells (Figure 4E).

### MiR-142 and miR-22 are inhibited both by Dox treatment and negatively correlated with Sox2OT-V7

**Figure 5:**
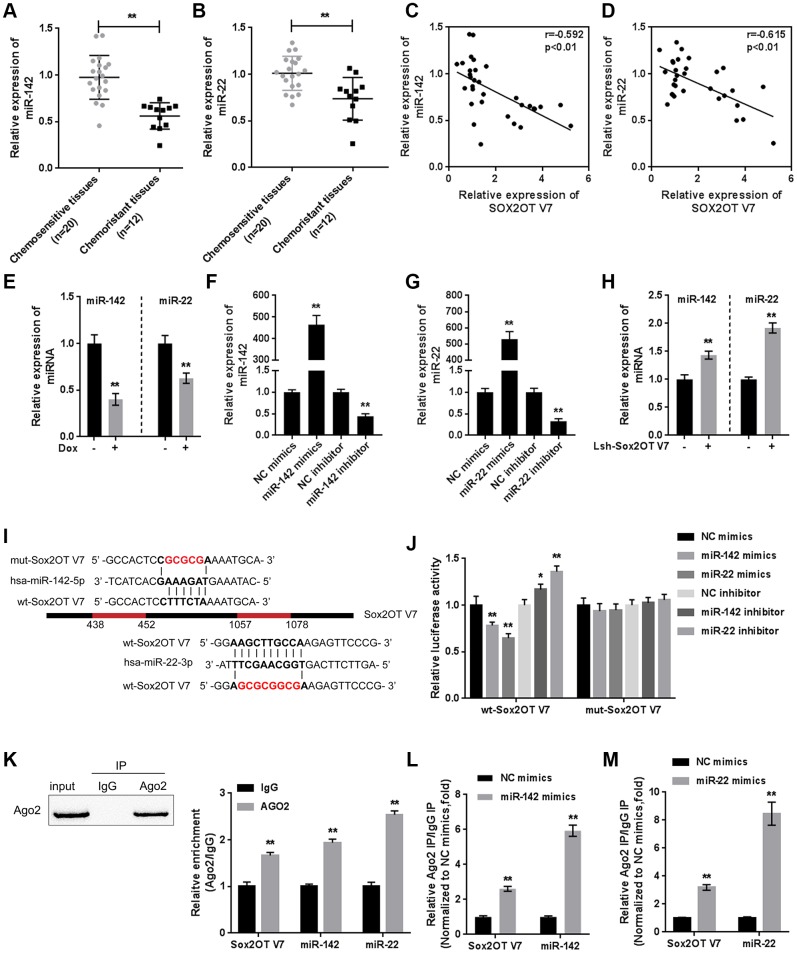
**miR-142 and miR-22 are inhibited by Dox treatment and negatively correlated with Sox2OT-V7.** (**A**, **B**) The expression of miR-142 and miR-22 in chemosensitive and chemoresistant OS tissues was determined by qPCR. (**C**, **D**) The correlation of Sox2OT-V7, miR-142, and miR-22 was analyzed by Pearson’s correlation analyses. (**E**) miR-142 and miR-22 expression in OS cells treated with Dox was determined by qPCR. (**F**, **G**) miR-142 and miR-22 overexpression and inhibition in U2OS cells were achieved by transfection of miR-142 and miR-22 mimics or inhibitor, as confirmed by qPCR. (**H**) miR-142 and miR-22 expression in Sox2OT-V7 silenced OS cells was determined by qPCR. (**I**) Predicted miR-142 and miR-22 binding sites in Sox2OT-V7. Wild-type and mutant-type Sox2OT-V7 reporter vectors containing wild-type or mutant-type miR-142 or miR-22 binding sites were constructed. (**J**) The above-described vectors were cotransfected in HEK293 cells with miR-142 or miR-22 mimics or inhibitor, and the luciferase activity was determined. (**K**) Association of Sox2OT-V7, miR-142, and miR-22 with AGO2 in HEK293 cells. Detection of AGO2 and IgG using immunoblotting assays. (**L**, **M**) RIP assay in HEK293 cells transfected with control miRNA (miR-NC) or miR-142 mimics or miR-22 mimics followed by real-time PCR to detect Sox2OT-V7 associated with AGO2. The data are presented as mean ± SD of three independent experiments. *P<0.05, **P<0.01.

As we have mentioned, miR-142 and miR-22 were selected for further experiments because of their correlations with Sox2OT-V7 and autophagy [13, 14]. miR-142 and miR-22 expression were both significantly lower in chemo-resistant OS tissues than in normal tissues (Figure 5A–5B). Consistent with data in the TCGA (Supplementary Figure 2), both miR-142 and miR-22, were negatively correlated with Sox2OT-V7 expression in tissue samples (Figure 5C, 5D). In OS cells, Dox treatment significantly inhibited the expression of miR-142 and miR-22 (Figure 5E).

To investigate the molecular function of miR-142 and miR-22, we achieved miR-142 and miR-22 expression by transfection of miR-142 or miR-22 mimics or inhibitor, as confirmed by qPCR (Figure 5F, 5G). In Sox2OT-V7-silenced U2OS cells, both miR-142 and miR-22 expression were significantly upregulated (Figure 5H). Next, a luciferase reporter assay was performed to validate the predicted binding of Sox2OT-V7, miR-142, and miR-22. Wild-type and mutant-type Sox2OT-V7 reporter vectors were constructed (Figure 5I) and cotransfected into HEK293 cells with miR-142 or miR-22 mimics or inhibitor and examined for luciferase activity. As shown in Figure 5J, the luciferase activity of wild-type vectors was significantly suppressed by miR-142 or miR-22 overexpression but enhanced by miR-142 or miR-22 inhibition. After mutating the predicted miR-142 or miR-22 binding sites, the alterations in the luciferase activity were abolished.

To further confirm the binding, RIP assays were performed. As shown in Figure 5K, Sox2OT-V7, miR-142, and miR-22 were associated with AGO2 in HEK293 cells. Sox2OT-V7, miR-142, and miR-22 levels were dramatically higher in the RNA derived from precipitated AGO2 protein than in that derived from IgG (Figure 5K). We also performed a RIP assay in HEK293 cells transfected with control miRNA (miR-NC) or miR-142 or miR-22 mimics followed by real-time PCR to detect the association of Sox2OT-V7 with AGO2; the results shown in Figure 5L and 5M confirmed the interaction between Sox2OT-V7 and miR-142 and between Sox2OT-V7 and miR-22.

### Dynamic effect of Sox2OT-V7, miR-142, and miR-22 on Dox-induced autophagy in U2OS cells

**Figure 6:**
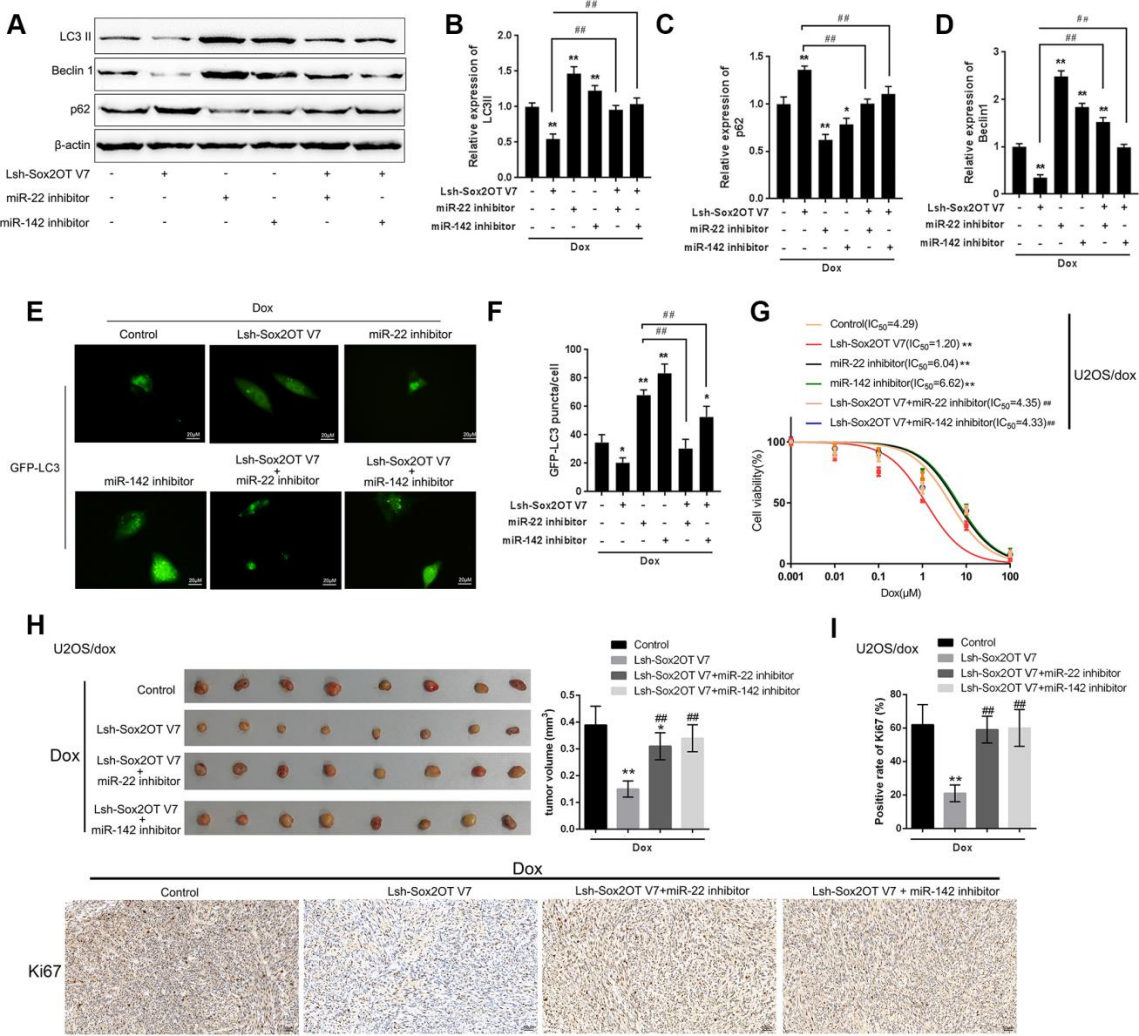
**Dynamic effect of Sox2OT-V7, miR-142, and miR-22 on Dox-induced autophagy in U2OS cells.** (**A**–**D**) OS cells were c-transfected with Lsh-Sox2OT-V7 and miR-142 inhibitor or miR-22 inhibitor upon Dox treatment and examined for the protein levels of LC3 II, Beclin 1, and p62 were examined. (**E**, **F**) U2OS cells with stable eGFP-LC3 expression were cotransfected with Lsh-Sox2OT-V7 and miR-142 inhibitor or miR-22 inhibitor, treated with Dox (5 μM) for 24 h, and examined for the formation of puncta by using a confocal microscope. Representative images are presented. (**G**) U2OS/Dox cells were assigned to six groups: control group, single Lsh-Sox2OT-V7 group, single miR-22 inhibitor group, single miR-142 inhibitor group, Lsh-Sox2OT-V7 + miR-22 inhibitor group, and Lsh-Sox2OT-V7 + miR-142 inhibitor group. Cells were transfected accordingly, treated with a series of concentrations of Dox (0.001, 0,01, 0.1, 1, 10, and 100 μM), and examined for the cell viability by MTT assay. (**H**) An *in vivo* tumor xenograft assay was performed by injecting U2OS/Dox cells that were not infected, infected with Lsh-Sox2OT-V7, transduced with Lsh-Sox2OT-V7 + miR-22 inhibitor, or transduced with Lsh-Sox2OT-V7 + miR-142 inhibitor under Dox treatment (n = 8 in each group). Tumors were shown and tumor volumes were detected. (**I**) Cell proliferation within the tumor was determined by IHC staining using an anti-Ki67 antibody. The data are presented as the mean ± SD of three independent experiments. *P<0.05, **P<0.01, compared to the control group; #*P*<0.05, ##*P*<0.01, compared to the Lsh-Sox2OT-V7 group.

After confirming the direct binding between Sox2OT-V7 and miR-142 and between Sox2OT-V7 and miR-22, we investigated the dynamic effect of Sox2OT-V7, miR-142, and miR-22 on Dox-induced autophagy in U2OS cells. miR-142 or miR-22 inhibition increased the protein levels of LC3II and Beclin 1 while decreased p62 protein under Dox treatment; the effect of miR-142 or miR-22 inhibition was partially reversed by 3-MA (Supplementary Figure 3A–3H). Next, U2OS cells were cotransfected with Lsh-Sox2OT-V7 and miR-142 or miR-22 inhibitor and the protein levels of autophagy markers and autophagy flux were examined. As revealed by immunoblotting, Dox-induced LC3II and Beclin 1 protein upregulation was partially attenuated by Sox2OT-V7 silencing but enhanced by miR-142 inhibition or miR-22 inhibition (Figure 6A–6D). Moreover, the effect of Sox2OT-V7 silencing was partially reversed by miR-142 inhibition or miR-22 inhibition (Figure 6A–6D). Furthermore, IF observation revealed similar results: miR-142 inhibition or miR-22 inhibition increased while Sox2OT-V7 silencing decreased the number of green puncta, the effect of Sox2OT-V7 silencing was also partially reversed by 142 inhibition or miR-22 inhibition (Figure 6E, 6F). Consistently, upon Dox treatment, Sox2OT-V7 silencing significantly reduced the IC_50_ value of U2OS/Dox cells to 1.20 μM, and miR-22 or miR-142 inhibition increased the IC50 values to 6.04 and 6.62 μM, respectively; the effects of Sox2OT-V7 silencing on U2OS/Dox cell viability were partially reversed by either miR-22 inhibition (IC_50_ = 4.35 μM) or miR-142 inhibition (IC_50_ = 4.33 μM) (Figure 6G). These data indicate that Sox2OT-V7 modulates Dox-induced autophagy in U2OS cells via its downstream targets miR-142 and miR-22, therefore affecting the chemoresistance of Dox-resistant OS cells to Dox.

To further confirm the *in vitro* findings, next, the *in vivo* effects of lncRNA Sox2OT-V7 silencing were examined in a xenograft mouse model derived from U2OS/Dox cells (not infected, infected with single Lsh-Sox2OT-V7, transduced with Lsh-Sox2OT-V7 + miR-22 inhibitor, or transduced with Lsh-Sox2OT-V7 + miR-142 inhibitor; n = 8). Under Dox treatment, lncRNA Sox2OT-V7 silencing significantly reduced the tumor volume (**P<0.01, Figure 6H); however, miR-22 inhibition or miR-142 inhibition significantly reversed the effects of Sox2OT-V7 silencing on tumor volumes upon Dox treatment (##*P*<0.01, Figure 6H). As also confirmed by IHC staining using an anti-Ki67 antibody, cell proliferation within the tumors was significantly reduced by lncRNA Sox2OT-V7 silencing under Dox treatment (**P<0.01, Figure 6I), while miR-22 inhibition or miR-142 inhibition significantly reversed the effects of Sox2OT-V7 silencing on cell proliferation within the tumors upon Dox treatment (##*P*<0.01, Figure 6I). These data indicate that lncRNA Sox2OT-V7 silencing in U2OS/Dox cells resensitized Dox-resistant cells to Dox treatment.

### MiR-22/miR-142 targets key autophagy proteins ATG5, ATG4A and ULK1

**Figure 7:**
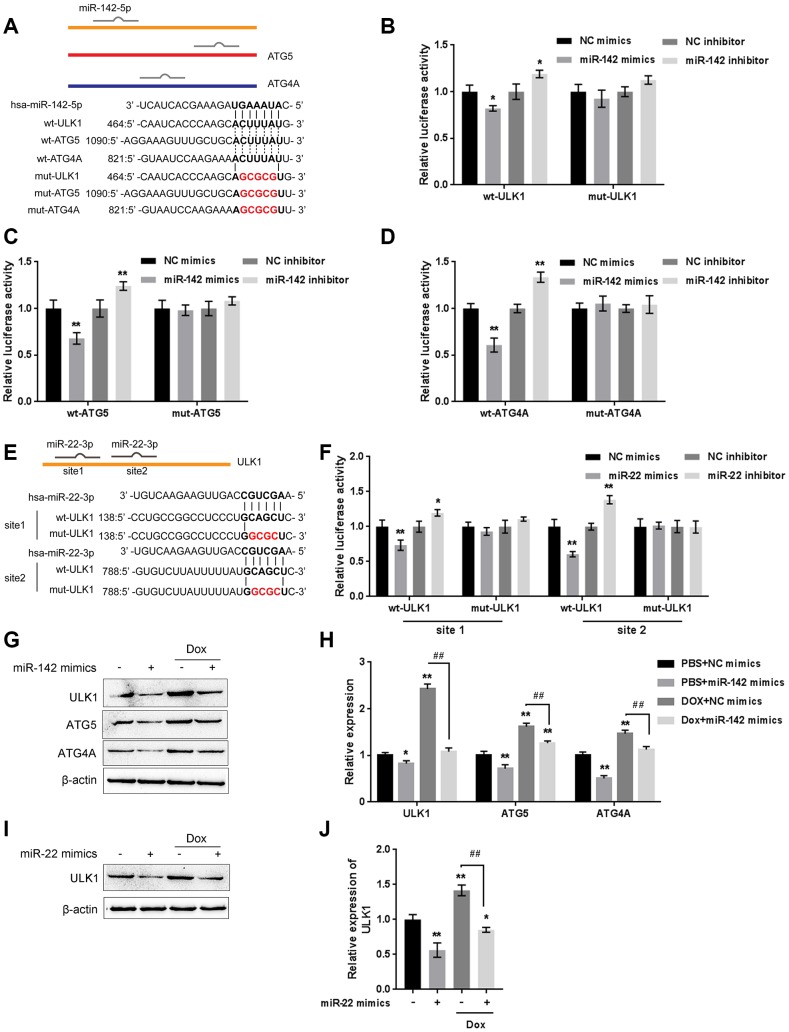
**miR-22/miR-142 targets the key autophagy proteins ATG5, ATG4A and ULK1.** (**A**) Predicted miR-142 binding sites in the ULK1, ATG4A, and ATG5 3'UTR. Wild-type and mutant-type ULK1, ATG4A, and ATG5 3'UTR reporter vectors containing wild-type or mutant-type miR-142 binding sites were constructed. (**B**–**D**) These vectors were co-transfected into HEK293 cells with miR-142 mimics or inhibitor, and the luciferase activity was determined. (**E**) Predicted miR-22 binding sites in ULK1. Wild-type and mutant-type ULK1 3'UTR reporter vectors containing wild- or mutant-type miR-22 binding sites were constructed. (**F**) These vectors were cotransfected into HEK293 cells with miR-22 mimics or inhibitor, and the luciferase activity was determined. (**G**, **H**) U2OS cells were transfected with miR-142 mimics in the presence or absence of Dox and examined for the protein levels of ULK1, ATG4A, and ATG5 were examined. (**I**, **J**) U2OS cells were transfected with miR-22 mimics in the presence or absence of Dox (5 μM), and the protein levels of ULK1 were examined. The data are presented as the mean ± SD of three independent experiments. *P<0.05, **P<0.01, compared to the control group; ##*P*<0.01, compared to the Dox group.

The kinase activity of ULK/Atg1 is required for autophagy. ULK phosphorylates Beclin-1 following amino acid withdrawal, and this phosphorylation step is crucial for the function of Beclin-1 in autophagy [17, 18]. Previously, miR-142-3p was reported to target ATG-1 (ATG16L1) [19]. Here, online tools predicted that miR-142 and miR-22 might target ULK and that miR-142 may target ATG4A and ATG5. To validate the predicted interactions, we performed luciferase reporter assays. Wild-type and mutant-type reporter vectors (wt- and mut-ULK1 3'UTR, ATG4A 3'UTR, and ATG5 3'UTR) containing wild-type or mutated predicted miR-142 or miR-22 binding sites were constructed (Figure 7A and 7E) and cotransfected into HEK293 cells with miR-142 or miR-22 mimics or inhibitor. As shown in Figure 7B–7D, and 7F, the luciferase activity of wild-type vectors was significantly suppressed by miR-142 or miR-22 mimics but enhanced by miR-142 or miR-22 inhibitor. After mutating the putative miR-142 or miR-22 binding sites, the alterations in luciferase activity were abolished. These data indicate that miR-142 targets ULK1, ATG4A, and ATG5 and that miR-22 targets ULK1.

Next, the effect of miR-142 and miR-22 on the above-described factors was investigated under Dox treatment. The protein levels of ULK1, ATG4A, and ATG5 were markedly decreased by miR-142 overexpression but increased by Dox treatment, and Dox-induced ULK1, ATG4A, and ATG5 upregulation was partially reversed by miR-142 overexpression (Figure 7G, 7H). Similarly, ULK1 protein was significantly decreased by miR-22 overexpression but increased by Dox treatment, and Dox-induced ULK1 upregulation was partially reversed by miR-22 overexpression (Figure 7I, 7J). These data indicate that miR-142 targets ULK1, ATG4A, and ATG5, and miR-22 targets ULK1 to regulate the expression of these critical autophagy-related factors upon Dox treatment.

### The Sox2OT-V7/miR-142/miR-22 axis modulates autophagy in U2OS cells by regulating autophagy-related genes

**Figure 8:**
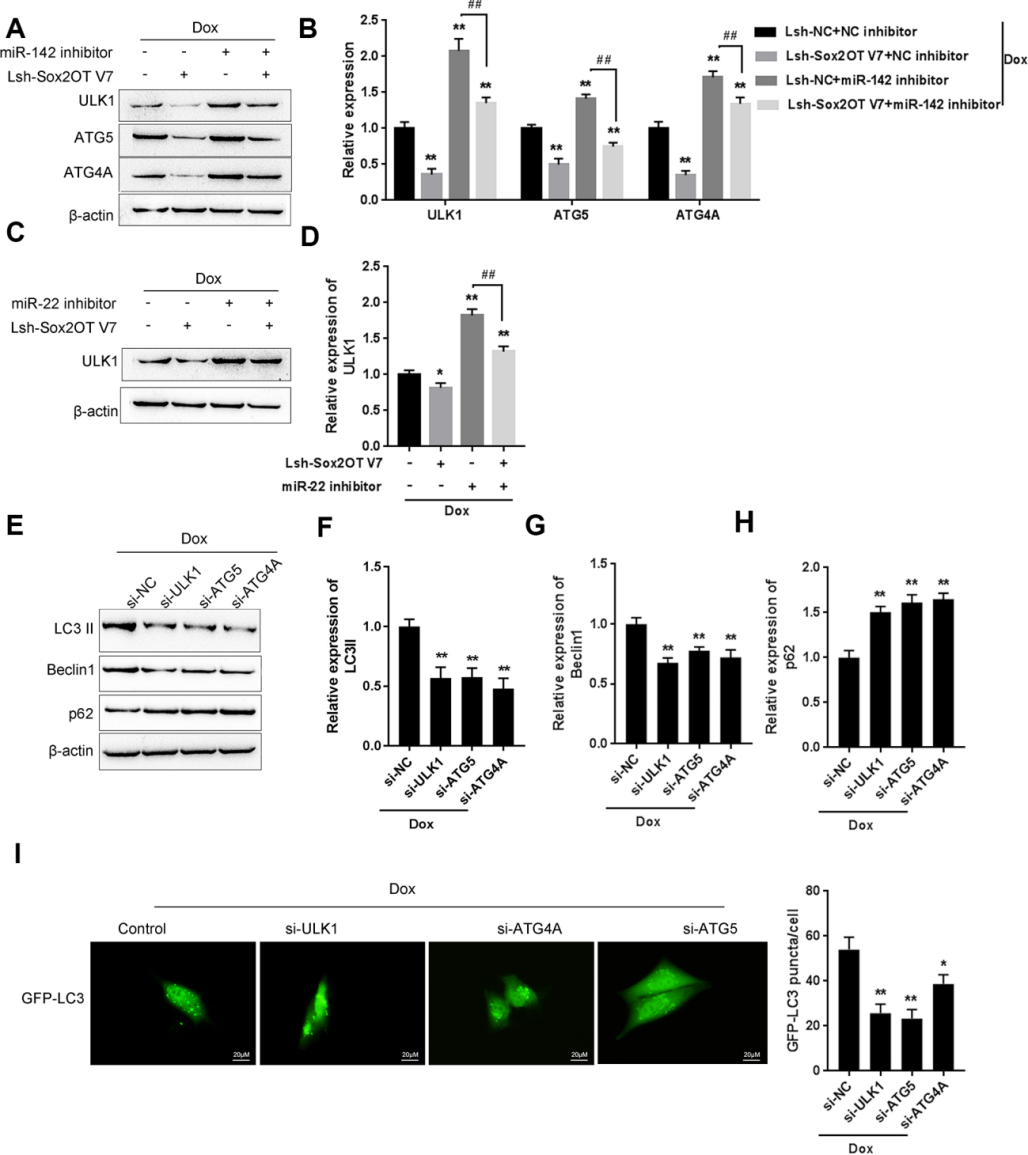
**The Sox2OT-V7/miR-142/miR-22 axis modulates autophagy in OS cells by regulating autophagy-related genes.** (**A**, **B**) U2OS cells were cotransfected with Lsh-Sox2OT-V7 and miR-142 inhibitor under Dox treatment and the protein levels of ULK1, ATG4A, and ATG5 were examined. (**C**, **D**) U2OS cells were cotransfected with Lsh-Sox2OT-V7 and miR-22 inhibitor under Dox treatment the protein levels of ULK1. (**E**–**H**) U2OS cells were transfected with si-ULK1, si-ATG4A, or si-ATG5 under Dox treatment and the protein levels of LC3 II, Beclin 1, and p62 were examined using immunoblotting. The data are presented as the mean ± SD of three independent experiments. *P<0.05, **P<0.01, compared to the control group; ##*P*<0.01, compared to the miR-142 inhibitor or miR-22 inhibitor group. (**I**) U2OS cells with stable eGFP-LC3 expression were transfected with si-ULK1, si-ATG4A, or si-ATG5 under Dox treatment, and the formation of puncta was examined by using a confocal microscope. Representative images are presented.

As we have mentioned, ULK1, ATG4A, and ATG5 are key autophagy-related factors [17, 18, 20]. Next, we investigated whether the Sox2OT-V7/miR-142/miR-22 axis could modulate autophagy in U2OS cells via these autophagy-related genes. U2OS cells were cotransfected with Lsh-Sox2OT-V7 and miR-142 inhibitor under Dox treatment, and the protein levels of ULK1, ATG4A, and ATG5 was examined. As shown in Figure 8A and 8B, the protein levels of ULK1, ATG4A, and ATG5 were significantly decreased by Sox2OT-V7 silencing but increased by miR-142 inhibition under Dox treatment, the effect of Sox2OT-V7 silencing was partially reversed by miR-142 inhibition. Next, U2OS cells were c-transfected with Lsh-Sox2OT-V7 and miR-22 inhibitor under Dox treatment and these factors were examined. Similarly, the protein levels of these autophagy-related genes were significantly decreased by Sox2OT-V7 silencing but increased by miR-22 inhibition under Dox treatment, and the effect of Sox2OT-V7 silencing was also partially reversed by miR-22 inhibition (Figure 8C, 8D).

To further confirm these findings, we evaluated the effect of ULK1, ATG4A, and ATG5 on the autophagy markers LC3, Beclin 1, and p62, and performed IF staining to monitor autophagy flux upon Dox treatment. The silencing of ULK1, ATG4A, and ATG5 was achieved by transfection of si-ULK1, si-ATG4A, and si-ATG5, as confirmed by immunoblotting (Supplementary Figure 4). As shown in Figure 8E–8H, Dox-induced LC3II and Beclin 1 upregulation was partially suppressed, while Dox-suppressed downregulation of p62 could be partially rescued by the silencing of ULK1, ATG4A, or ATG5. For IF observation, the green puncta were reduced by the silencing of ULK1, ATG4A, or ATG5 (Figure 8I), indicating that Dox-induced autophagy in U2OS cells was suppressed by the silencing of ULK1, ATG4A, or ATG5. Since the Sox2OT-V7/miR-142/miR-22 axis regulated the expression of ULK1, ATG4A, and ATG5 in U2OS cells, it can be concluded that the Sox2OT-V7/miR-142/miR-22 axis modulates autophagy in U2OS cells by regulating the autophagy-related genes ULK1, ATG4A, and ATG5.

### Expression of ULK1, ATG4A, and ATG5, and correlations with the Sox2OT-V7/miR-142/miR-22 axis in tissue samples

**Figure 9:**
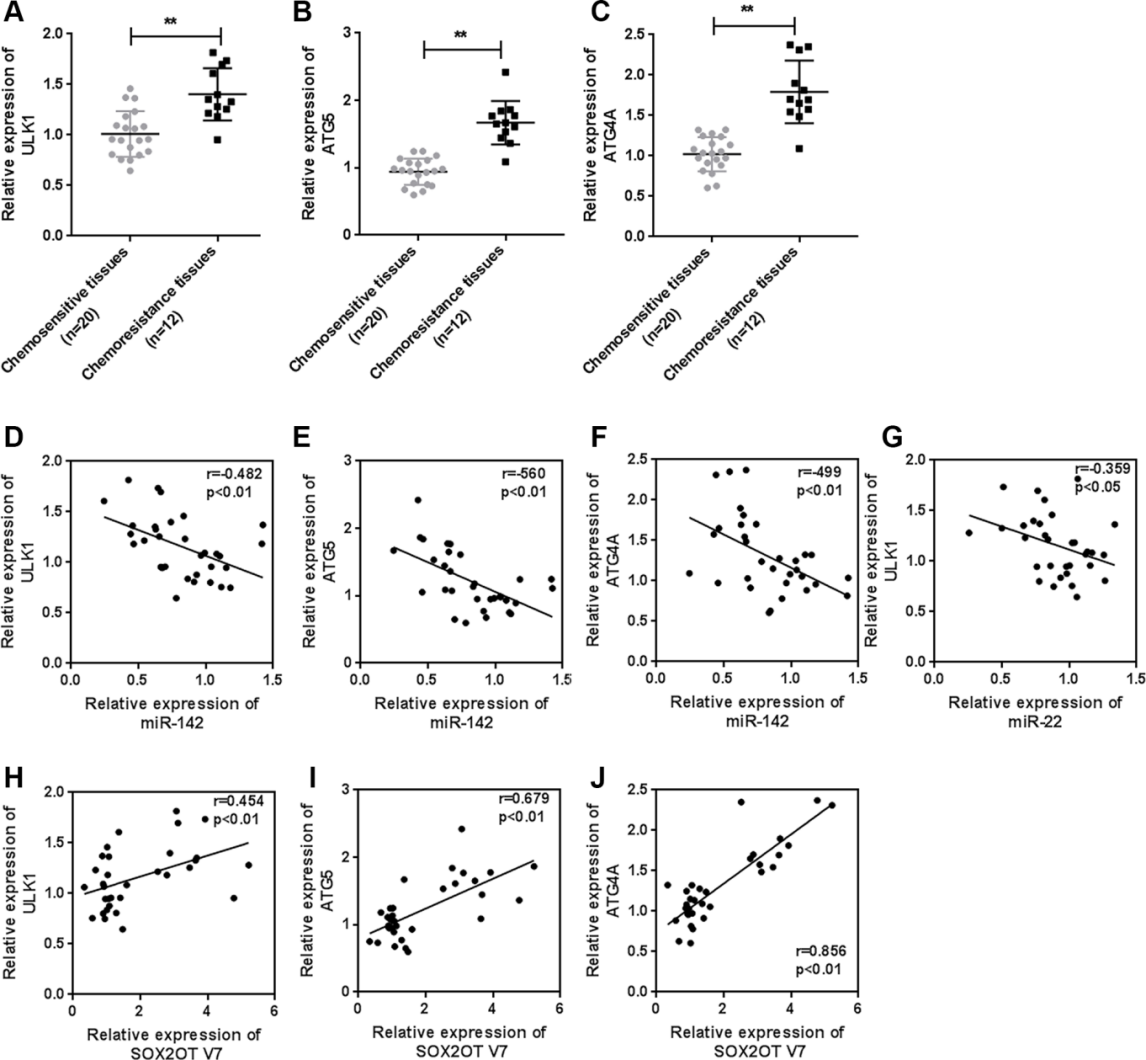
**Expression of ULK1, ATG4A, and ATG5, and correlations with the Sox2OT-V7/miR-142/miR-22 axis in tissue samples.** (**A**–**C**) The expression of ULK1, ATG4A, and ATG5 in 20 chemosensitive and 12 chemoresistant OS tissue samples was examined by qPCR. (**D**–**J**) The correlation of Sox2OT-V7, miR-142, miR-22, ULK1, ATG4A, and ATG5 was analyzed by Pearson’s correlation analysis. The data are presented as the mean ± SD of three independent experiments. *P<0.05, **P<0.01.

To further confirm the above findings, we examined the expression of ULK1, ATG4A, and ATG5 in tissue samples. The expression of ULK1, ATG4A, and ATG5 was higher in chemoresistant OS tissues than in chemosensitive tissues (Figure 9A–9C). MiR-142 was negatively correlated with ULK1, ATG4A, and ATG5 (Figure 9D–9F). MiR-22 was negatively correlated with ULK1 (Figure 9G). Sox2OT-V7 was positively correlated with ULK1, ATG4A, and ATG5 (Figure 9H–9J).

## DISCUSSION

**Figure 10:**
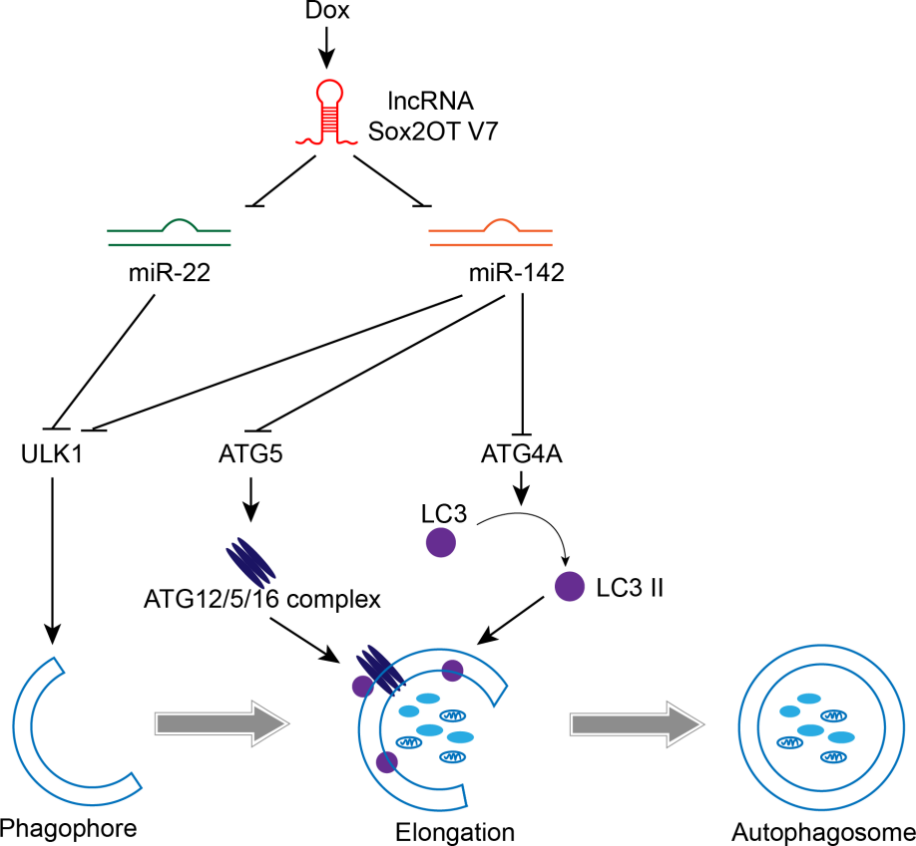
**A mechanistic schematic diagram showing the Sox2OT-V7/miR-142/miR-22 axis modulating the autophagy in OS cells via the autophagy-related genes, ULK1, ATG4A, and ATG5.**

In the present study, we revealed that Sox2OT-V7 expression is upregulated in OS tissues, particularly in chemoresistant OS tissues, and in OS cell lines compared to control. Dox treatment simultaneously induces autophagy and Sox2OT-V7 expression in U2OS cells, while Dox-induced autophagy can be partially attenuated by Sox2OT-V7 silencing. Moreover, Sox2OT-V7 directly targets miR-142 and miR-22 to inhibit their expression, and the effect of Sox2OT-V7 silencing on U2OS cell autophagy can be partially reversed by miR-142 or miR-22 inhibition. Finally, miR-142 directly targets ULK1, ATG4A, and ATG5, while miR-22 directly targets ULK1 to inhibit the expression of the target genes; the Sox2OT-V7/miR-142/miR-22 axis modulates autophagy in U2OS cells by regulating ULK1, ATG4A, and ATG5 (Figure 10).

Autophagy is a self-digestion and recycling mechanism used by cells to survive harsh environmental conditions. Autophagy and/or autophagy dysregulation are speculated to play various, sometimes conflicting roles in the development and progression of neoplasia [21, 22]. The chemotherapeutic agent Dox induces autophagy in human osteosarcoma cell lines [23]. Autophagy contributes to chemoresistance in canine appendicular osteosarcoma and adding an autophagy inhibitor to the standard of care has the potential to improve outcome [15]. In the present study, we constructed a Dox-resistant U2OS cell subtype, U2OS/Dox, and consistently observed that autophagy inhibitor 3-MA could resensitize U2OS/Dox to Dox treatment.

The dysregulation of Sox2OT-V7 has been widely reported in cancers, including esophageal squamous cell carcinoma [24], hepatocellular carcinoma [25], gastric cancer [26], cholangiocarcinoma [27], and OS [11, 28]. In the present study, we also observed significantly upregulated Sox2OT-V7 expression in OS cell lines and tissue samples, particularly chemoresistant tissue samples. In our previous study, we demonstrated that Dox-induced pro-survival autophagy was suppressed by decreasing Sox2OT-V7; therefore, the growth inhibition of Dox was improved [11]. Zhao D et al found that Dox treatment induced increased expression levels of cleaved caspase-3 and LC3, but reduced levels of p62 in osteosarcoma cells, and inhibition of autophagy notably enhanced the effects of Dox [23]. In the present study, we also found that Dox induced the ratio of LC3II/I and Beclin 1 protein while decreasing p62 protein, therefore causing autophagy flux. The upregulation of Sox2OT-V7 was also induced by Dox treatment. More importantly, the effect of Dox treatment on autophagy markers and autophagy flux was significantly attenuated by Sox2OT-V7 silencing, indicating that Sox2OT-V7 silencing may improve OS chemoresistance by acting on LC3, a core factor in the process of autophagy [29]. To further investigate the speculation that Sox2OT-V7 silencing may improve OS chemoresistance by modulating autophagy, in vitro and in vivo experiments were performed. As expected, knocking down Sox2OT-V7 resensitize U2OS/Dox cells to Dox treatment *in vitro*, similar to treatment with the autophagy inhibitor 3-MA.

Accumulating evidence suggests that lncRNAs act as miRNA sponges and inhibit miRNA functions [12]. Recently, lncRNA-associated ceRNA networks were revealed to play an essential role in many types of human cancer, including OS [30]. For example, lncRNA TUG1 promotes cell proliferation and suppresses apoptosis in osteosarcoma by regulating the miR-212-3p/FOXA1 axis [31]. LncRNA HOXA-AS2 promotes migration and invasion by acting as a ceRNA of miR-520c-3p in OS [32]. Here, we searched the TCGA database for miRNAs negatively correlated with Sox2OT-V7 and found that miR-142 and miR-22 may be direct downstream targets of Sox2OT-V7. In chemoresistant tissue samples, miR-142 and miR-22 expression were both significantly downregulated. Furthermore, consistent with online tool prediction, Sox2OT-V7 directly targets miR-142 and miR-22 to inhibit their expression. As for the molecular functions, miR-142 and miR-22 inhibition enhanced Dox-induced autophagy in OS cells, and partially reversed the effect of Sox2OT-V7 silencing on LC3II/I, Beclin 1, p62, and autophagy flux under Dox treatment. These data indicate that the Sox2OT-V7/miR-142/miR-22 axis modulates Dox-induced autophagy in OS cells. More importantly, knocking down Sox2OT-V7 enhanced the suppressive effects of Dox on U2OS/Dox cell-derived tumor growth and the cell proliferation within the tumor. In contrast, miR-22 inhibition or miR-142 inhibition attenuated the effects of Sox2OT-V7 silencing on Dox-induced suppression of tumor growth. In other words, Sox2OT-V7 silencing resensitized tumors to Dox treatment, while miR-22 inhibition or miR-142 inhibition enhanced the drug-resistance of tumors.

Both miR-142 and miR-22 are regarded as inhibitory miRNAs in autophagy. miR-22 regulates 5-FU sensitivity by inhibiting autophagy and promoting apoptosis in colorectal cancer cells [13]. By targeting HMGB1, miR-22 inhibits HMGB1-mediated autophagy in OS cells, thereby acting as a tumor suppressor [33, 34]. miR-142-3p overexpression increases the chemosensitivity of non-small-cell lung cancer by inhibiting HMGB1-mediated autophagy [35]. MiR-142-3p inhibits autophagy by targeting ATG16L1 in thymic-derived regulatory T cells [14, 19]. As predicted by online tools and validated by luciferase reporter assays, miR-142 directly targets ULK1, ATG4A, and ATG5, and miR-22 directly targets ULK1.

There are five subgroups of “core” Atg proteins in mammals: the ULK1 protein kinase complex [36], Vps34-beclin1 class III PI3-kinase complex [39], Atg9-WIPI-1 complex [38–40], Atg12 conjugation system [41, 42], and LC3 conjugation system [43, 44]. Autophagy is impaired without any of these “core” Atg gene products, indicating that a sequential reaction of many protein complexes, including kinases, phosphatases, lipids, and ATP-dependent conjugation, are indispensable for the whole process of autophagy. In the present study, miR-142 targets ULK1/ATG4A/ATG5, and miR-22 targets ULK1 to inhibit expression of the target genes. More importantly, Sox2OT-V7 silencing decreases, while miR-142 or miR-22 inhibition increases the protein levels of ULK1, ATG4A, and ATG5; Sox2OT-V7 silencing-suppressed ULK1, ATG4A, and ATG5 expression is partially rescued by miR-142 or miR-22 inhibition, indicating that Sox2OT-V7 serves as a ceRNA for miR-142 and miR-22 to counteract miR-142 or miR-22-mediated suppression of downstream core Atg genes. Consistently, ULK1, ATG4A, and ATG5 silencing partially attenuates Dox-induced alterations in the LC3II/I ratio, Beclin 1, and p62 proteins, as well as autophagy flux, indicating that the Sox2OT-V7/miR-142/miR-22 axis modulates Dox-induced autophagy via downstream “core” Atg genes.

## CONCLUSIONS

lncRNA Sox2OT-V7 promotes Dox-induced autophagy and chemoresistance in OS via tumor-suppressive miR-142/miR-22 and downstream core Atg genes (Figure 10). Upon further *in vivo* validation, these findings may provide new directions for combating OS chemoresistance to Dox-based therapies.

## MATERIALS AND METHODS

### Clinical samples and chemoresistance evaluation

A total of 32 paired OS and nontumorous tissue samples were obtained from patients who received the same chemotherapy regimen before surgery and underwent complete resection surgery at The Second Xiangya Hospital with the approval of the Ethics Committee of The Second Xiangya Hospital, and written informed consent was obtained from all the patients. All the resected specimens were stored at -80 °C. According to the Huvos scoring system [45], the patients were classified as good responders (non-chemo-resistant) and poor responders (chemo-resistant).

### Cell lines and cell culture

Four OS cell lines, MNNG/HOS Cl #5 (ATCC® CRL-1547™), MG63 (ATCC® CRL-1427™), U2OS (ATCC® HTB-96™), and Saos-2 (ATCC® HTB-85™), and a normal osteoblast cell line, hFOB (ATCC® CRL-11372™), were obtained from ATCC (Manassas, VA, USA). The cells were maintained in DMEM supplemented with 10% FBS, 25 mM hydroxyethyl piperazine ethane sulfonic acid buffer, 100 U/mL penicillin, and 100 μg/mL streptomycin in a humidified atmosphere of 5% CO_2_ at 37 °C.

### Construction of Sox2OT-V7 silencing lentivirus

The sh-Sox2OT-V7 sequence was synthesized by Beijing Genomics Institute (Beijing, China). The sh-Sox2OT-V7 and lentiviral vector PGMLV-6395 were enzyme-digested with BamHI/EcoRI. Ligation was performed to construct Sox2OT-V7 silenced lentivirus recombinant plasmid. The following lentivirus package was performed by Auragene Biotech (Changsha, China).

### Quantitative RT-PCR

Total RNA was extracted from cells using TRIzol Reagent (Invitrogen, Carlsbad, CA, USA) following the protocols, and then the RNA was reverse transcribed using the PrimeScript RT Master Mix Perfect Real-Time kit (TaKaRa, Dalian, China) to obtain the cDNA. A real-time PCR assay was performed under the reaction conditions described in our previous study [11] using cDNA as the template. After the reaction, the data were subjected to statistical analysis. Relative gene expression was calculated using the 2^-ΔΔCT^ method, and GAPDH (for mRNA) or U6 (for miRNA) served as an internal control. The primer sequence was listed in Supplementary Table 1.

### Immunoblotting

Cells were lysed in RIPA buffer with protease inhibitors and phosphate inhibitors. Protein was loaded onto an SDS-PAGE mini-gel and transferred onto a PVDF membrane. The blots were probed with the following primary antibodies: anti-LC3 (ab48394, Abcam, Cambridge, MA, USA), anti-Beclin 1 (ab207612, Abcam), anti-p62 (ab56416, Abcam), anti-ULK1 (ab167139, Abcam), anti-ATG5 (ab108327, Abcam), anti-ATG4A (ab108322, Abcam) Next, the blots were probed with the HRP-conjugated secondary antibody. Signals were visualized using ECL Substrates (Millipore, USA). GAPDH served as the loading control.

### Immunohistochemical (IHC) analysis

Immunohistochemistry (IHC) was performed according to the indirect immunoperoxidase method. In brief, following deparaffinization, hydration and blockage of endogenous peroxidase, the specimens were incubated for 20 min with 10% nonfat milk in PBS to block specific sites and then individually incubated at 4°C overnight with the following primary antibodies: anti-LC3 (1:2000, ab48394, Abcam), anti-Beclin 1 antibody (1:400, ab207612, Abcam), and anti-Ki67 antibody (ab15580, Abcam). After rinsing, the slides were washed, and the sections were incubated with biotinylated goat anti-rabbit secondary antibody for 30 min at room temperature, and then incubated using the Vectastain ABC-AP kit (Vector Laboratories, Burlingame, CA, USA) for 30 min. Finally, the sections were washed and incubated with DAB substrate for 2-8 min.

### Autophagy detection

Autophagy levels were detected using immunofluorescence staining and transmission electron microscopy. U2OS cells were transduced with eGFR-LC3 lentivirus (Hanbio, China) for 24 h. Then, transfected cells were treated with DOX or rapamycin (Sigma) for 24 h. Cells were then fixed with 4% PFA for 30 min and washed with PBS 3 times. The immunofluorescent images were obtained by using a confocal fluorescence microscope (BX50; Olympus, Japan) and representative images are presented. Transmission electron microscopy detection of autophagic vacuoles was performed as previously described [46].

### *In vivo* tumor xenograft

U2OS/Dox (Dox-resistant) cells (1 × 10^7^) transduced with lentivirus (Lsh-NC or Lsh-Sox2OT-V7) were subcutaneously injected into the nude mice (SLAC Experimental Animal Center, Changsha, China). Two weeks after tumor formation, mice were received intraperitoneal injection. There were four experimental groups: control, Lsh-Sox2OT-V7, Lsh-Sox2OT-V7+miR-22 inhibitor and Lsh-Sox2OT-V7+miR-142 inhibitor. The mice in all groups were intraperitoneally injected with Dox (2 mg/kg, once a week for 4 weeks). Tumor volume was measured every 3 days after the formation of the xenografted tumor. Forty-two days after the inoculation of U2OS/Dox cells, the mice were sacrificed by euthanasia. All experimental procedures in the current study were approved by the Central South University Animal Care Committee.

### Luciferase reporter assay

Fragments of Sox2OT-V7 and of the ATG4A 3'UTR, ATG5 3'UTR, and ULK 3'UTR were amplified by PCR and cloned downstream of the Renilla psiCHECK2 vector (Promega, Madison, WI, USA), the resulting vectors were named wt-Sox2OT-V7, wt-ATG4A 3'UTR, wt-ATG5 3'UTR, and wt-ULK 3'UTR. To generate mutant reporter vectors, the seed regions of the above-described fragments were mutated to remove all complementarity to miR-142 or miR-22 and named mut-Sox2OT-V7, mut-ATG4A 3'UTR, mut-ATG5 3'UTR, and mut-ULK 3'UTR. HEK293 cells (ATCC) were seeded into a 24-well plate overnight and cotransfected with the indicated vectors and miR-142 or miR-22 mimics or inhibitor. Luciferase assays were performed 48 h after transfection using the Dual-Luciferase Reporter Assay System (Promega). Renilla luciferase activity was normalized to firefly luciferase activity for each transfected well.

### RNA immunoprecipitation (RIP)

RIP was performed using Magna RIP RNA-Binding Protein Immunoprecipitation Kit (17-700, Millipore) following the protocols. RNA for *in vitro* experiments was transcribed using T7 High YieldRNA Synthesis Kit (E2040S, NEB) according to the manufacturer’s instructions. Sox2OT-V7, miR-142, and miR-22 levels in the immunoprecipitates were measured by qRT-PCR.

### Statistical analysis

Data was expressed as the means ± SD of at least three independent experiments and statistically analyzed by one-way analysis of variance (ANOVA) followed by Tukey's multiple comparison test or independent sample *t*-test using the SPSS Statistics 17.0 software. The level of significance was assessed by thresholds of *P* < 0.05 and *P* < 0.01.

## Supplementary Material

Supplementary Figures

Supplementary Tables

## References

[1] Ottaviani G, Robert RS, Huh WW, Palla S, Jaffe N. Sociooccupational and physical outcomes more than 20 years after the diagnosis of osteosarcoma in children and adolescents: limb salvage versus amputation. Cancer. 2013; 119:3727–36. 10.1002/cncr.28277 23907996 PMC3842284

[2] Huang J, Ni J, Liu K, Yu Y, Xie M, Kang R, Vernon P, Cao L, Tang D. HMGB1 promotes drug resistance in osteosarcoma. Cancer Res. 2012; 72:230–38. 10.1158/0008-5472.CAN-11-2001 22102692

[3] Chen L, Ye HL, Zhang G, Yao WM, Chen XZ, Zhang FC, Liang G. Autophagy inhibition contributes to the synergistic interaction between EGCG and doxorubicin to kill the hepatoma Hep3B cells. PLoS One. 2014; 9:e85771. 10.1371/journal.pone.0085771 24465696 PMC3897495

[4] Jin F, Wang Y, Li M, Zhu Y, Liang H, Wang C, Wang F, Zhang CY, Zen K, Li L. MiR-26 enhances chemosensitivity and promotes apoptosis of hepatocellular carcinoma cells through inhibiting autophagy. Cell Death Dis. 2017; 8:e2540. 10.1038/cddis.2016.461 28079894 PMC5386370

[5] Sun R, Shen S, Zhang YJ, Xu CF, Cao ZT, Wen LP, Wang J. Nanoparticle-facilitated autophagy inhibition promotes the efficacy of chemotherapeutics against breast cancer stem cells. Biomaterials. 2016; 103:44–55. 10.1016/j.biomaterials.2016.06.038 27376558

[6] Wang X, Wang XL, Chen HL, Wu D, Chen JX, Wang XX, Li RL, He JH, Mo L, Cen X, Wei YQ, Jiang W. Ghrelin inhibits doxorubicin cardiotoxicity by inhibiting excessive autophagy through AMPK and p38-MAPK. Biochem Pharmacol. 2014; 88:334–50. 10.1016/j.bcp.2014.01.040 24522112

[7] Jaeger PA, Wyss-Coray T. All-you-can-eat: autophagy in neurodegeneration and neuroprotection. Mol Neurodegener. 2009; 4:16. 10.1186/1750-1326-4-16 19348680 PMC2679749

[8] Gibbings D, Mostowy S, Jay F, Schwab Y, Cossart P, Voinnet O. Selective autophagy degrades DICER and AGO2 and regulates miRNA activity. Nat Cell Biol. 2012; 14:1314–21. 10.1038/ncb2611 23143396 PMC3771578

[9] Chang Z, Huo L, Li K, Wu Y, Hu Z. Blocked autophagy by miR-101 enhances osteosarcoma cell chemosensitivity in vitro. ScientificWorldJournal. 2014; 2014:794756. 10.1155/2014/794756 25013866 PMC4072053

[10] Wang Z, Liu Z, Wu S. Long non-coding RNA CTA sensitizes osteosarcoma cells to doxorubicin through inhibition of autophagy. Oncotarget. 2017; 8:31465–77. 10.18632/oncotarget.16356 28415557 PMC5458222

[11] Wang W, Chen D, Zhu K. SOX2OT variant 7 contributes to the synergistic interaction between EGCG and Doxorubicin to kill osteosarcoma via autophagy and stemness inhibition. J Exp Clin Cancer Res. 2018; 37:37. 10.1186/s13046-018-0689-3 29475441 PMC6389193

[12] Paraskevopoulou MD, Hatzigeorgiou AG. Analyzing mirna-lncrna interactions. Methods Mol Biol. 2016; 1402:271–86. 10.1007/978-1-4939-3378-5_21 26721498

[13] Zhang H, Tang J, Li C, Kong J, Wang J, Wu Y, Xu E, Lai M. MiR-22 regulates 5-FU sensitivity by inhibiting autophagy and promoting apoptosis in colorectal cancer cells. Cancer Lett. 2015; 356:781–90. 10.1016/j.canlet.2014.10.029 25449431

[14] Zhang K, Chen J, Zhou H, Chen Y, Zhi Y, Zhang B, Chen L, Chu X, Wang R, Zhang C. PU.1/microRNA-142-3p targets ATG5/ATG16L1 to inactivate autophagy and sensitize hepatocellular carcinoma cells to sorafenib. Cell Death Dis. 2018; 9:312. 10.1038/s41419-018-0344-0 29472524 PMC5833744

[15] Schott CR, Ludwig L, Mutsaers AJ, Foster RA, Wood GA. The autophagy inhibitor spautin-1, either alone or combined with doxorubicin, decreases cell survival and colony formation in canine appendicular osteosarcoma cells. PLoS One. 2018; 13:e0206427. 10.1371/journal.pone.0206427 30372478 PMC6205606

[16] Horie R, Nakamura O, Yamagami Y, Mori M, Nishimura H, Fukuoka N, Yamamoto T. Apoptosis and antitumor effects induced by the combination of an mTOR inhibitor and an autophagy inhibitor in human osteosarcoma MG63 cells. Int J Oncol. 2016; 48:37–44. 10.3892/ijo.2015.3227 26530936 PMC4734606

[17] Nazarko VY, Zhong Q. ULK1 targets Beclin-1 in autophagy. Nat Cell Biol. 2013; 15:727–28. 10.1038/ncb2797 23817237 PMC4442023

[18] Russell RC, Tian Y, Yuan H, Park HW, Chang YY, Kim J, Kim H, Neufeld TP, Dillin A, Guan KL. ULK1 induces autophagy by phosphorylating Beclin-1 and activating VPS34 lipid kinase. Nat Cell Biol. 2013; 15:741–50. 10.1038/ncb2757 23685627 PMC3885611

[19] Lu Y, Gao J, Zhang S, Gu J, Lu H, Xia Y, Zhu Q, Qian X, Zhang F, Zhang C, Shen H, Hippen KL, Blazar BR, et al. miR-142-3p regulates autophagy by targeting ATG16L1 in thymic-derived regulatory T cell (tTreg). Cell Death Dis. 2018; 9:290. 10.1038/s41419-018-0298-2 29459719 PMC5833855

[20] Fernández AF, López-Otín C. The functional and pathologic relevance of autophagy proteases. J Clin Invest. 2015; 125:33–41. 10.1172/JCI73940 25654548 PMC4382236

[21] White E, DiPaola RS. The double-edged sword of autophagy modulation in cancer. Clin Cancer Res. 2009; 15:5308–16. 10.1158/1078-0432.CCR-07-5023 19706824 PMC2737083

[22] Mathew R, Karantza-Wadsworth V, White E. Role of autophagy in cancer. Nat Rev Cancer. 2007; 7:961–67. 10.1038/nrc2254 17972889 PMC2866167

[23] Zhao D, Yuan H, Yi F, Meng C, Zhu Q. Autophagy prevents doxorubicin-induced apoptosis in osteosarcoma. Mol Med Rep. 2014; 9:1975–81. 10.3892/mmr.2014.2055 24639013

[24] Wu Y, Chen X, Liang Y, Li J, Zhang K, Dai L, Guan X, Wang K, Bai Y. Overexpression of long non-coding RNA SOX2OT promotes esophageal squamous cell carcinoma growth. Cancer Cell Int. 2018; 18:76. 10.1186/s12935-018-0570-7 29849506 PMC5970475

[25] Sun J, Wei X, Xu L. Upregulation of lncRNA Sox2ot indicates a poor prognosis for patients with hepatocellular carcinoma and promotes cell invasion. Oncol Lett. 2018; 16:1189–95. 10.3892/ol.2018.8725 29963193 PMC6019957

[26] Qu F, Cao P. Long noncoding RNA SOX2OT contributes to gastric cancer progression by sponging miR-194-5p from AKT2. Exp Cell Res. 2018; 369:187–96. 10.1016/j.yexcr.2018.05.017 29782828

[27] Li Z, Li J, Ji D, Leng K, Xu Y, Huang L, Jiang X, Cui Y. Overexpressed long noncoding RNA Sox2ot predicts poor prognosis for cholangiocarcinoma and promotes cell proliferation and invasion. Gene. 2018; 645:131–36. 10.1016/j.gene.2017.12.017 29246536

[28] Wang Z, Tan M, Chen G, Li Z, Lu X. LncRNA SOX2-OT is a novel prognostic biomarker for osteosarcoma patients and regulates osteosarcoma cells proliferation and motility through modulating SOX2. IUBMB Life. 2017; 69:867–76. 10.1002/iub.1681 28960757

[29] Tanida I. Autophagy basics. Microbiol Immunol. 2011; 55:1–11. 10.1111/j.1348-0421.2010.00271.x 21175768

[30] Xie L, Yao Z, Zhang Y, Li D, Hu F, Liao Y, Zhou L, Zhou Y, Huang Z, He Z, Han L, Yang Y, Yang Z. Deep RNA sequencing reveals the dynamic regulation of miRNA, lncRNAs, and mRNAs in osteosarcoma tumorigenesis and pulmonary metastasis. Cell Death Dis. 2018; 9:772. 10.1038/s41419-018-0813-5 29991755 PMC6039476

[31] Xie C, Chen B, Wu B, Guo J, Cao Y. LncRNA TUG1 promotes cell proliferation and suppresses apoptosis in osteosarcoma by regulating miR-212-3p/FOXA1 axis. Biomed Pharmacother. 2018; 97:1645–53. 10.1016/j.biopha.2017.12.004 29793327

[32] Wang Y, Zhang R, Cheng G, Xu R, Han X. Long non-coding RNA HOXA-AS2 promotes migration and invasion by acting as a ceRNA of miR-520c-3p in osteosarcoma cells. Cell Cycle. 2018; 17:1637–48. 10.1080/15384101.2018.1489174 30081707 PMC6133314

[33] Li X, Wang S, Chen Y, Liu G, Yang X. miR-22 targets the 3′ UTR of HMGB1 and inhibits the HMGB1-associated autophagy in osteosarcoma cells during chemotherapy. Tumour Biol. 2014; 35:6021–28. 10.1007/s13277-014-1797-0 24609901

[34] Guo S, Bai R, Liu W, Zhao A, Zhao Z, Wang Y, Wang Y, Zhao W, Wang W. miR-22 inhibits osteosarcoma cell proliferation and migration by targeting HMGB1 and inhibiting HMGB1-mediated autophagy. Tumour Biol. 2014; 35:7025–34. 10.1007/s13277-014-1965-2 24752578

[35] Chen Y, Zhou X, Qiao J, Bao A. Mir-142-3p overexpression increases chemo-sensitivity of nsclc by inhibiting hmgb1-mediated autophagy. Cell Physiol Biochem. 2017; 41:1370–82. 10.1159/000467896 28427045

[36] Mizushima N. The role of the Atg1/ULK1 complex in autophagy regulation. Curr Opin Cell Biol. 2010; 22:132–39. 10.1016/j.ceb.2009.12.004 20056399

[37] Liang XH, Jackson S, Seaman M, Brown K, Kempkes B, Hibshoosh H, Levine B. Induction of autophagy and inhibition of tumorigenesis by beclin 1. Nature. 1999; 402:672–76. 10.1038/45257 10604474

[38] Young AR, Chan EY, Hu XW, Köchl R, Crawshaw SG, High S, Hailey DW, Lippincott-Schwartz J, Tooze SA. Starvation and ULK1-dependent cycling of mammalian Atg9 between the TGN and endosomes. J Cell Sci. 2006; 119:3888–900. 10.1242/jcs.03172 16940348

[39] Webber JL, Young AR, Tooze SA. Atg9 trafficking in Mammalian cells. Autophagy. 2007; 3:54–56. 10.4161/auto.3419 17102588

[40] Proikas-Cezanne T, Ruckerbauer S, Stierhof YD, Berg C, Nordheim A. Human WIPI-1 puncta-formation: a novel assay to assess mammalian autophagy. FEBS Lett. 2007; 581:3396–404. 10.1016/j.febslet.2007.06.040 17618624

[41] Mizushima N, Noda T, Yoshimori T, Tanaka Y, Ishii T, George MD, Klionsky DJ, Ohsumi M, Ohsumi Y. A protein conjugation system essential for autophagy. Nature. 1998; 395:395–98. 10.1038/26506 9759731

[42] Mizushima N, Sugita H, Yoshimori T, Ohsumi Y. A new protein conjugation system in human. The counterpart of the yeast Apg12p conjugation system essential for autophagy. J Biol Chem. 1998; 273:33889–92. 10.1074/jbc.273.51.33889 9852036

[43] Kabeya Y, Mizushima N, Ueno T, Yamamoto A, Kirisako T, Noda T, Kominami E, Ohsumi Y, Yoshimori T. LC3, a mammalian homologue of yeast Apg8p, is localized in autophagosome membranes after processing. EMBO J. 2000; 19:5720–28. 10.1093/emboj/19.21.5720 11060023 PMC305793

[44] Ichimura Y, Kirisako T, Takao T, Satomi Y, Shimonishi Y, Ishihara N, Mizushima N, Tanida I, Kominami E, Ohsumi M, Noda T, Ohsumi Y. A ubiquitin-like system mediates protein lipidation. Nature. 2000; 408:488–92. 10.1038/35044114 11100732

[45] Ferrari S, Ruggieri P, Cefalo G, Tamburini A, Capanna R, Fagioli F, Comandone A, Bertulli R, Bisogno G, Palmerini E, Alberghini M, Parafioriti A, Linari A, et al. Neoadjuvant chemotherapy with methotrexate, cisplatin, and doxorubicin with or without ifosfamide in nonmetastatic osteosarcoma of the extremity: an Italian sarcoma group trial ISG/OS-1. J Clin Oncol. 2012; 30:2112–18. 10.1200/JCO.2011.38.4420 22564997

[46] Tang D, Kang R, Livesey KM, Kroemer G, Billiar TR, Van Houten B, Zeh HJ 3rd, Lotze MT. High-mobility group box 1 is essential for mitochondrial quality control. Cell Metab. 2011; 13:701–11. 10.1016/j.cmet.2011.04.008 21641551 PMC3293110

