# Supplementary material for: LncRNA Sox2OT-V7 promotes doxorubicin-induced autophagy and chemoresistance in osteosarcoma via tumor-suppressive miR-142/miR-22
Source: Aging (Albany NY). 2020 Apr 16;12(8):6644–66. doi: 10.18632/aging.103004 (PMC7202483; doi:10.18632/aging.103004)
Supplement: Supplementary Figures [file aging-12-103004-s002..pdf]

SUPPLEMENTARY FIGURES

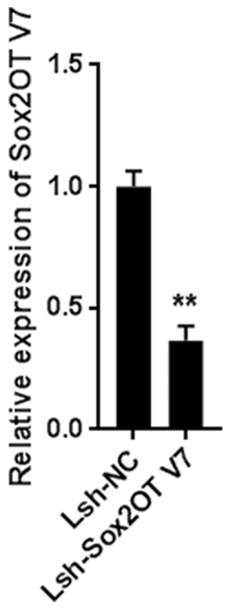

Supplementary Figure 1. The knockdown effect of Sox2OT-V7 was determined by QPCR.

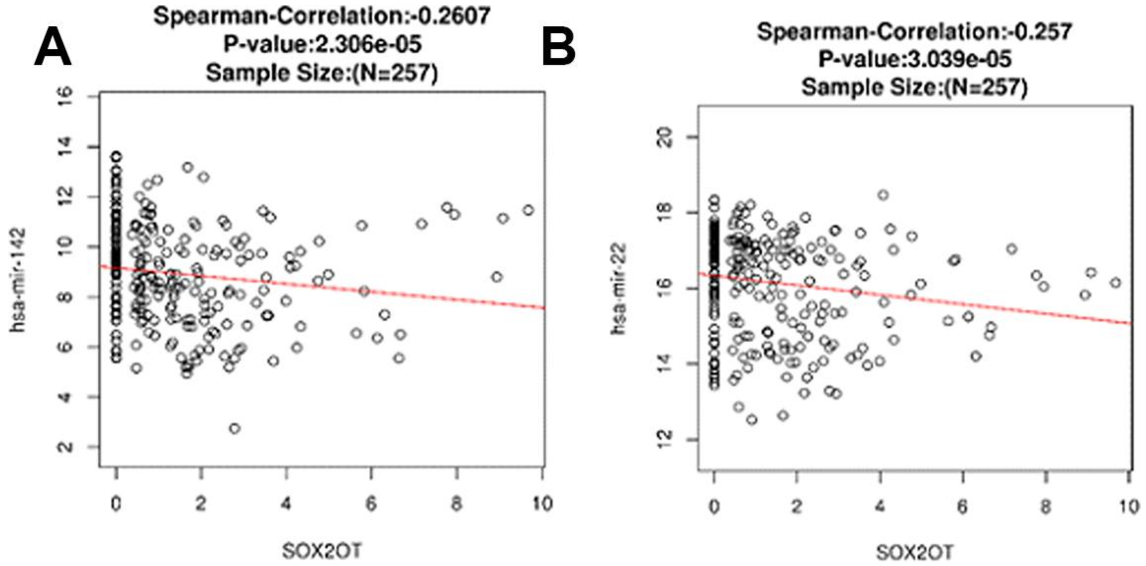

Supplementary Figure 2. The correlation of SOX2OT, miR-142 (**A**) miR-22 (**B**) expression in the TCGA database.

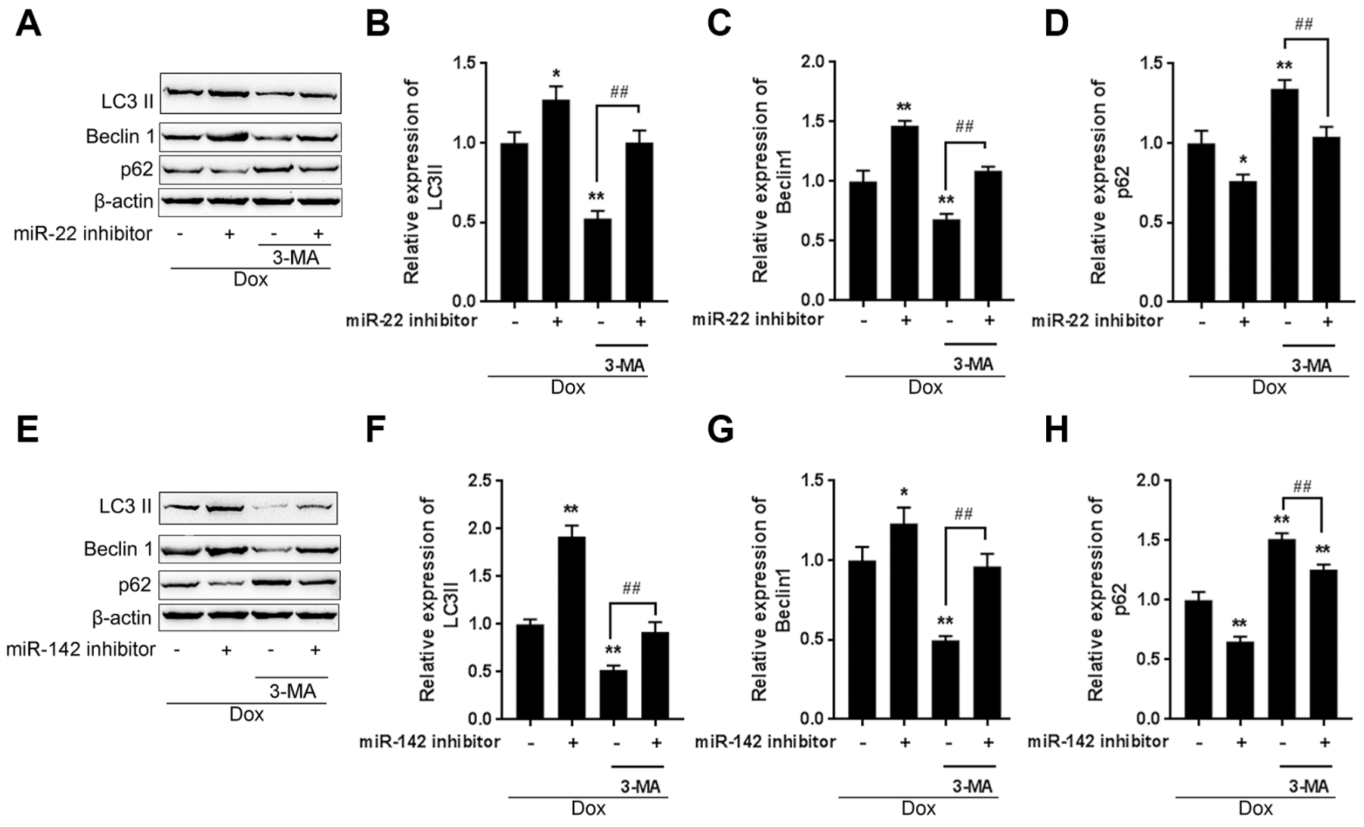

Supplementary Figure 3. The effect of miR-142 and miR-22 on Dox-induced autophagy. (**A**–**D**) U2OS cells were transfected with miR-142 inhibitor and co-tread with Dox (5 μM) and 3-MA (5 mM) for 24 h and the protein levels of LC3 II, Beclin 1 and p62 were examined. (**E**–**H**) U2OS cells were transfected with miR-22 inhibitor and cotreated with Dox and 3-MA for 24 h and the protein levels of LC3 II, Beclin 1 and p62 were examined.

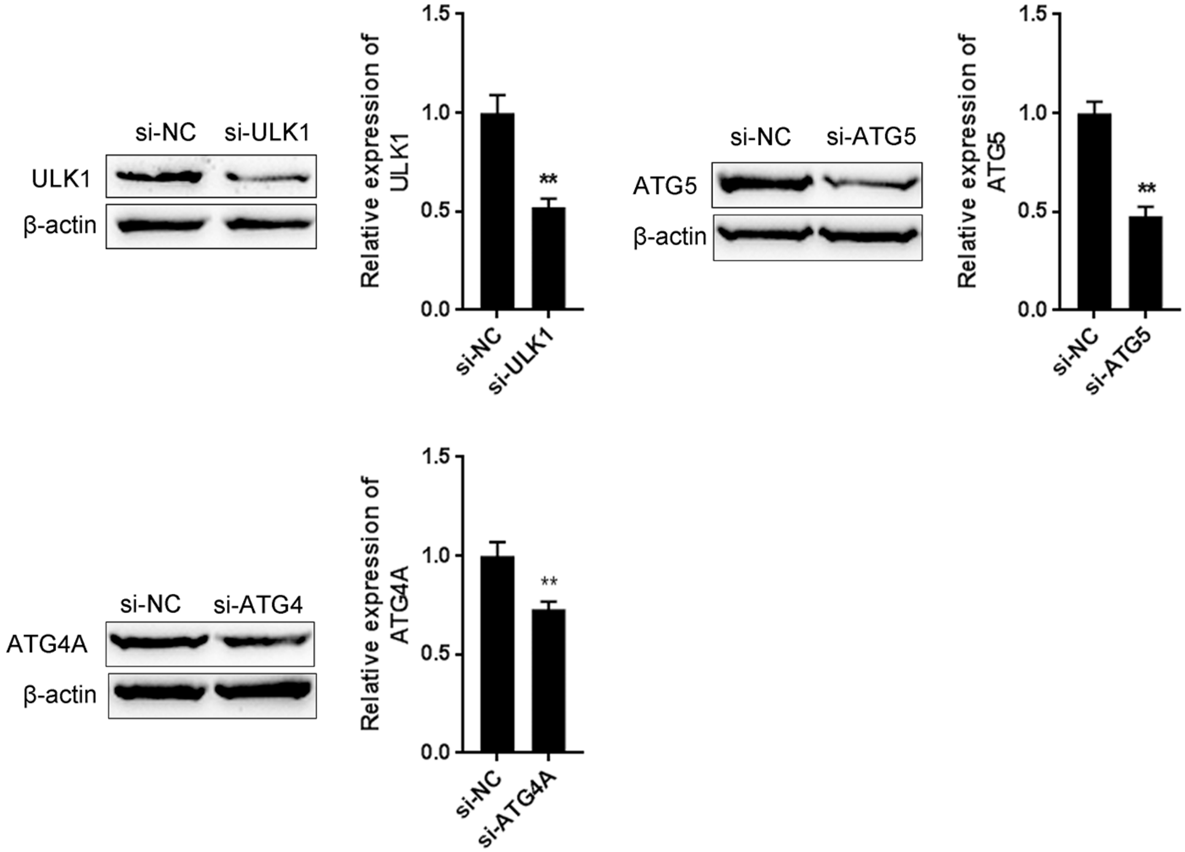

Supplementary Figure 4. The knockdown effects of ULK1, ATG5 and ATG4A were confirmed by immunoblotting.
