# Supplementary material for: LncRNA Sox2OT-V7 promotes doxorubicin-induced autophagy and chemoresistance in osteosarcoma via tumor-suppressive miR-142/miR-22
Source: Aging (Albany NY). 2020 Apr 16;12(8):6644–66. doi: 10.18632/aging.103004 (PMC7202483; doi:10.18632/aging.103004)
Supplement: Supplementary Tables [file aging-12-103004-s001..pdf]

SUPPLEMENTARY TABLES

Supplementary Table 1. The primer sequence.

Name	Forward 5'-3'	Reverse5'-3'
SOX2OT V7	TCTGTTCAGTATTTGGAAGAAAG	ACATTATTTCTAAGTTGGATATGTC
Beclin 1	CCATGCAGGTGAGCTTCGT	GAATCTGCGAGAGACACCATC
Mir-142-5P	RT:GTCGTATCCAGTGCGTGTCGTGGAGTC GGCAATTGCACTGGATACGACAGTAGT F:GCCGCATAAAGTAGAAAGC	CAGTGCGTGTCGTGGA
Mir-22-3P	RT:GTCGTATCCAGTGCGTGTCGTGGAGTC GGCAATTGCACTGGATACGACACAGTT F:GCAAGCTGCCAGTTGAAG	CAGTGCGTGTCGTGGA
ULK1	GGCAAGTTCGAGTTCTCCCG	CGACCTCCAAATCGTGCTTCT
ATG5	AAAGATGTGCTTCGAGATGTGT	CACTTTGTCAGTTACCAACGTCA
ATG4A	TGCTGGTTGGGGATGTATGC	GCGTTGGTATTCTTTGGGTTGT
b-actin	TTCCAGCCTTCCTTCCTGGG	TTGCGCTCAGGAGGAGCAAT
U6	CTCGCTTCGGCAGCACA	AACGCTTCACGAATTTGCGT
Mir-142 mimics	CAUAAAGUAGAAAGCACUACU	UAGUGCUUUCUACUUUAUGTT
MiR-22 mimics	AAGCUGCCAGUUGAAGAACUGU	AGUUCUUCAACUGGCAGCUUTT
Mir-142 inhibitor	AGUAGUGCUUUCUACUUUAUG	
MiR-22 inhibitor	ACAGUUCUUCAACUGGCAGCUU	
NC mimics	UUCUCCGAACGUGUCACGUTT	ACGUGACACGUUCGGAGAATT
NC inhibitor	CAGUACUUUUGUGUAGUACAA	
Si-NC	UUCUCCGAACGUGUCACGUTT	ACGUGACACGUUCGGAGAATT
Si- ULK1	GAGGCAGUUCUUUGUUCAATT	UUGAACAAAGAACUGCCUCTT
Si-ATG5	GAUUUGUAUUUCUGAUUAATT	UUAAUCAGAAAUACAAAUCTT
Si-ATG4A	GGUUCUUGUUAAAUUAUUUTT	AAAUAAUUUAACAAGAACCTT
Lsh-SOX2OT V7	GATCCGGATAGGCCTCACTTACAAGACTC GAGTCTTGTAAGTGAGGCCTATCCTTTTTG	AATTCAAAAAGGATAGGCCTCACTTACAAG ACTCGAGTCTTGTAAGTGAGGCCTATCCG

Supplementary Table 2. miRNAs negatively correlated with SOX2-OT expression in OS tissue from TCGA data.

Name	Statistic	P-value	FDR (BH)
hsa-mir-1228	-0.24872	5.55E-05	0.003067
hsa-mir-1245	-0.21408	0.00055	0.017018
hsa-mir-142	-0.26074	2.31E-05	0.001983
hsa-mir-150	-0.24011	0.000101	0.004675
hsa-mir-1537	-0.22076	0.000363	0.012202
hsa-mir-155	-0.25456	3.64E-05	0.002492
hsa-mir-185	-0.24972	5.16E-05	0.003067
hsa-mir-21	-0.21484	0.000524	0.016912
hsa-mir-22	-0.25702	3.04E-05	0.002353
hsa-mir-223	-0.2042	0.000994	0.028486
hsa-mir-3614	-0.32554	9.32E-08	1.80E-05
hsa-mir-511-1	-0.33101	5.49E-08	1.42E-05
hsa-mir-511-2	-0.33603	3.35E-08	1.30E-05
hsa-mir-548j	-0.20161	0.001155	0.031932
hsa-mir-942	-0.22245	0.000326	0.011463
